# Accurate predictions of population-level changes in sequence and structural properties of HIV-1 Env using a volatility-controlled diffusion model

**DOI:** 10.1371/journal.pbio.2001549

**Published:** 2017-04-06

**Authors:** Orlando DeLeon, Hagit Hodis, Yunxia O’Malley, Jacklyn Johnson, Hamid Salimi, Yinjie Zhai, Elizabeth Winter, Claire Remec, Noah Eichelberger, Brandon Van Cleave, Ramya Puliadi, Robert D. Harrington, Jack T. Stapleton, Hillel Haim

**Affiliations:** 1 Department of Microbiology, Carver College of Medicine, University of Iowa, Iowa City, Iowa, United States of America; 2 Center for AIDS Research (CFAR) at the University of Washington, Seattle, Washington, United States of America; 3 Veterans Affairs Medical Center, Iowa City, Iowa, United States of America; Imperial College London, United Kingdom of Great Britain and Northern Ireland

## Abstract

The envelope glycoproteins (Envs) of HIV-1 continuously evolve in the host by random mutations and recombination events. The resulting diversity of Env variants circulating in the population and their continuing diversification process limit the efficacy of AIDS vaccines. We examined the historic changes in Env sequence and structural features (measured by integrity of epitopes on the Env trimer) in a geographically defined population in the United States. As expected, many Env features were relatively conserved during the 1980s. From this state, some features diversified whereas others remained conserved across the years. We sought to identify “clues” to predict the observed historic diversification patterns. Comparison of viruses that cocirculate in patients at any given time revealed that each feature of Env (sequence or structural) exists at a defined level of variance. The in-host variance of each feature is highly conserved among individuals but can vary between different HIV-1 clades. We designate this property “volatility” and apply it to model evolution of features as a linear diffusion process that progresses with increasing genetic distance. Volatilities of different features are highly correlated with their divergence in longitudinally monitored patients. Volatilities of features also correlate highly with their population-level diversification. Using volatility indices measured from a small number of patient samples, we accurately predict the population diversity that developed for each feature over the course of 30 years. Amino acid variants that evolved at key antigenic sites are also predicted well. Therefore, small “fluctuations” in feature values measured in isolated patient samples accurately describe their potential for population-level diversification. These tools will likely contribute to the design of population-targeted AIDS vaccines by effectively capturing the diversity of currently circulating strains and addressing properties of variants expected to appear in the future.

## Author summary

HIV-1 is the causative agent of the global AIDS pandemic. The envelope glycoproteins (Envs) of HIV-1 constitute a primary target for antibody-based vaccines. However, the diversity of Envs in the population limits the potential efficacy of this approach. Accurate estimates of the range of variants that currently infect patients and those expected to appear in the future will likely contribute to the design of population-targeted immunogens. We found that different properties (features) of Env have different propensities for small “fluctuations” in their values among viruses that infect patients at any given time point. This propensity of each feature for in-host variance, which we designate “volatility”, is conserved among patients. We apply this parameter to model the evolution of features (in patients and population) as a diffusion process driven by their “diffusion coefficients” (volatilities). Using volatilities measured from a few patient samples from the 1980s, we accurately predict properties of viruses that evolved in the population over the course of 30 years. The diffusion-based model described here efficiently captures evolution of phenotypes in biological systems controlled by a dominant random component.

## Introduction

HIV-1 is the primary etiologic agent of the global AIDS pandemic. Soon after identification of HIV-1 in the early 1980s, the tremendous sequence diversity of circulating strains was appreciated [1, 2]. The genetic diversity of HIV-1 has posed a major obstacle to development of an efficacious vaccine. Several factors contribute to the sequence heterogeneity of this virus. Mutations are frequently introduced in the viral genome during replication by the error-prone reverse transcriptase enzyme [3–7]. In addition, HIV-1 has a high propensity for recombination during coinfection of a cell by two different isolates [8–12]. The high rate of viral replication (10^10^–10^12^ new virions can be generated daily) increases the appearance of sequence variants [13, 14]. Persistence of the newly formed variants in the host is determined by the selective pressures exerted on the different virus components. Of the proteins encoded by HIV-1, the envelope glycoproteins (Envs) show the greatest degree of in-host and between-host diversity [15].

The HIV-1 Envs are contained on the surface of the viral particle and function as a membrane-fusing machine that mediates entry into host cells [16, 17]. Env is composed of a surface subunit (gp120) and a transmembrane subunit (gp41) [18]. HIV-1 infection of the host is most frequently initiated by a single virus [19–22]. From this founder state, the virus replicates to form multiple quasispecies, which elicit formation of antibodies that can bind to Env and neutralize infectivity of virions [23, 24]. However, frequent mutations in Env allow emergence of escape variants that contain changes in the antibody-targeted epitopes. Such variants can then persist in the infected individual. Thus, antibody pressure applied by the host defines properties of circulating Envs [14, 25, 26]. A second type of pressure applied on Env is the requirement to effectively fuse with host cells. This pressure is dynamic, since availability of susceptible cells can alter during the course of infection and in different body compartments [27–35]. As a consequence of selection, there is continuous replacement of circulating viral lineages in the host, associated with increased divergence of viral quasispecies [29, 36–42]. The balance between forces that increase sequence diversity (i.e., random mutations and recombinations) and the selective forces that act to contain diversity determines the pattern of virus evolution in the infected host [29, 40, 43, 44].

Multiple host factors affect the population-level evolution of HIV-1, including the humoral and cellular immune responses [45–47] and antiretroviral treatment regimens [48, 49]. Several studies have examined historic population-level trends in genomic properties of HIV-1 [50–53]. In addition, changes in sensitivity of circulating strains to the humoral and cellular immune responses have been studied [54–56]. Changes are often attributed to population-level adaptation of the virus to the host immune response and to the fitness pressures applied. Nevertheless, for most properties of Env (sequence and structural), the basis for the observed changes is not understood. Why are certain properties altered whereas others remain conserved over the years? Are these trends sufficiently stable to allow us to predict the range of variants that will evolve in the future? The ability to capture the current diversity of phenotypes in the population and to predict properties of future variants will likely improve the efficacy of population-targeted immunogens.

To achieve the above goals, we conducted a combined cross-sectional (population-level) and longitudinal study in a defined geographic location in the United States. Two types of properties (features) were examined: (i) integrity of Env epitopes recognized by broadly neutralizing antibodies, and (ii) sequence characteristics (e.g., identity of individual amino acids at key antigenic sites or the length and charge of Env segments). We found that each Env feature is maintained at a defined level of variance among strains cocirculating in the same host at any given time, which is conserved among different individuals. We designate this property of each feature as its “volatility index.” Based on this parameter, we modeled the changes in sequence and structural features of Env in the patient and population as a linear diffusion process, which progresses with increasing genetic diversity. In-host volatility is highly correlated with the longitudinal divergence of features in patients over time. A strong relationship also exists between the volatility of each feature and its diversity in the population. Based on volatility indices measured in a small set of patient samples collected during the 1980s, we accurately predict the diversity of features that developed during the next three decades. Therefore, volatility and its translation patterns into population diversity explain many of the historic changes in Env features during the course of the epidemic. The ability to predict clade-specific patterns of change through limited patient sampling will likely contribute to the tailoring of AIDS vaccines to structural properties of Envs circulating within specific populations and the changes expected to occur during defined timeframes in the future.

## Results

### Design of a combined population-level and longitudinal study to characterize evolution of HIV-1 Env features in a defined geographic location

Several studies indicate that population-level changes have occurred in HIV-1 properties over the course of the AIDS pandemic [54–56]. To identify “clues” that could help us predict future population-level changes in sequence and structural properties of Env, we conducted a comprehensive study in a geographically-defined region of the US. Plasma samples provided to the University of Iowa HIV Clinic between 1985 and 2012 were used for isolation of the *env* gene from circulating viruses. A total of 371 Envs from 113 Iowa City patients were examined. For 101 patients, one plasma sample was available (these samples are designated below as cross-sectional). In addition, 12 patients provided longitudinal samples, collected over the course of 2–11 y. We also isolated 177 Envs from longitudinal plasma samples of 14 patients from the University of Washington Center for AIDS Research (CFAR) repository in Seattle (designated as UW samples). From each plasma sample, we amplified *env* genes of individual viruses by the single genome amplification (SGA) method [20, 57]. Amplification products were cloned into a vector that allows expression of the Env protein. To focus our studies on Envs of potentially transmissible viruses, we measured the ability of each Env to mediate entry into cells (see Materials and methods section). Further analyses were performed only for fusion-competent Envs. To avoid direct effects of antiretroviral therapy (ART) on Env structure or function, none of the patients were treated by entry inhibitors during or prior to plasma sample collection. Phylogenetic relationships between Envs isolated for this work are shown in S1 Fig. All primary data, GenBank accession numbers, and the amino acid sequence alignment are provided in S1, S2 and S3 Data. All Envs from Iowa City and Seattle belong to clade B viruses, except Envs from Iowa City patients IC.798 and IC.999, which are from clades AD and A, respectively.

For each Env, we examined the integrity of defined epitopes by measuring recognition by specific probes using a cell-based ELISA system [58, 59]. The assay involves expression of full-length trimeric cleaved Envs on the surface of human osteosarcoma (HOS) cells and measurement of probe binding [60]. A panel of 11 broadly neutralizing monoclonal antibodies (mAbs) that recognize well-defined epitopes in antigenically dominant regions of Env was selected as structural probes. Such antibodies constitute the primary specificities in sera with broadly neutralizing activity [61, 62]. Distinct but overlapping epitopes were chosen for improved characterization of the structural layout of these regions. Carbohydrate binding antibodies PGT121 and PGT126 share a critical glycosylation site at position 332 [63] and partly compete with mAb 2G12 that targets mannose glycans on gp120 [64, 65]. This glycosylation site is highly accessible on the trimer and is considered a “supersite of vulnerability” for HIV-1 neutralization [66]. The mAbs PG9 and PG16 target overlapping, trimer-dependent epitopes [67]. The mAbs 2F5 and 10E8 target the N- and C-terminal domains of the gp41 membrane-proximal ectodomain region (MPER), respectively [68–71]. The 10E8 epitope is conserved among diverse HIV-1 clades whereas the 2F5 epitope shows some intra- and inter-clade diversity. Several probes that target the conserved, antigenically dominant CD4-binding site were tested, including mAbs b12, VRC03, and CD4-Ig, which contains two copies of CD4 linked to the Fc region of human IgG1 [58]. The mAb 39F binds to the V3 variable loop of gp120 [72], which shows clade-specific levels of solvent exposure [73] that we sought to characterize.

Binding of probes describes the integrity of their target epitopes and is expressed as a percent of probe binding to the control AD8 Env, which contains all epitopes tested in this study. Data are normalized for the level of Env expression using saturating concentrations of CD4-Ig [58]. The output of the binding assay spans a range of approximately five orders of magnitude. Since changes in epitope integrity are measured by fold-changes in probe binding efficiency, we minimized the confounding effects of the very high and low values by correcting the log_10_-transformed data with a logistic function (see Materials and methods section and S2 Fig).

### Different features of Env show distinct patterns of diversification in the population over the past three decades

**Fig 1:**
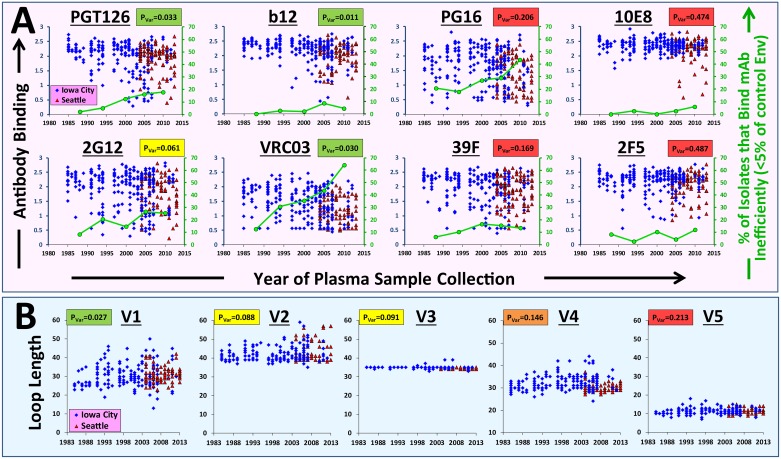
Structural and sequence features of HIV-1 Env present different patterns of historic change in the population. Historic changes in antigenic features **(A)** and length of the gp120 variable loops **(B)** in Envs isolated from samples collected in Iowa City (113 patients) and Seattle (14 patients). Each patient is represented by two isolates that reflect the range of feature values detected in the tested plasma sample. To examine changes in epitope integrity, we sectioned the three-decade time frame into 5–6 y periods. For each period, we quantified the percent of Envs that bind the probe inefficiently (marked by green circles), which is defined as less than 5% of probe binding to the control AD8 Env. To compare between feature variance in Period1 (1985–1991) and Period3 (2005–2012), we applied Levene’s test. The *p*-value for the null hypothesis of equal variance is labeled P_var_ and is highlighted in a color that describes its statistical significance (green, high; red, low). Changes in antigenicity features were tested using data from 27 patients from Period1 and 30 patients from Period3. Changes in variable loop lengths were tested using 32 and 31 patients from Period1 and Period3, respectively. All antigenicity and segmental features are shown in S3 and S4 Figs. Data underlying this figure can be found in S2 Data.

We examined historic changes in integrity of the above epitopes in viruses from Iowa City samples collected between 1985 and 2012. The epitopes tested were present in most Envs from the early part of the epidemic (1985–1991, designated herein Period1). However, during subsequent years, multiple isolates appeared with lower binding efficiencies, representing loss of epitope integrity (see Fig 1A and complete set of mAbs in S3 Fig). To quantify the historic changes in these features, we compared their population diversity during Period1 and Period3 (2005–2012) using Levene’s test for equality of variances (see details of statistical tools in the Materials and methods section). Different patterns of historic changes were observed for the epitopes. For example, PGT126, b12, and 10E8 show relatively low (and similar) levels of diversity during the early part of the epidemic. However, PGT126 and b12 gradually increased in their diversity from Period1 to Period3 whereas limited changes occurred in 10E8 (compare *p*-values of Levene’s test, labeled P_var_ in Fig 1A). Quantification of the percentage of Envs with very low or no binding of each probe showed that integrity of the 10E8, b12, and PGT126 epitopes was similar in the population during the 1980s (labeled by green circles in Fig 1A). However, the PGT126 epitope was gradually lost over the years whereas 10E8 and b12 were less affected. The epitopes of mAbs VRC03, 39F, and 2F5 were also similarly distributed in the population during the 1980s. However, VRC03 was then mostly eliminated (~70% of isolates from the latest time period do not contain this epitope) whereas the epitopes of 39F and 2F5 were retained in most circulating isolates.

We also examined historic changes in sequence features of the Env variable loops, including length, charge density (i.e., total charge per amino acid loop length), density of potential N-linked glycosylation sites (PNGSs), and mean loop hydropathy score (based on the Black and Mould scale [74]). For definition of Env features and segment boundaries, see Materials and methods section. Macroarchitectural (segmental) properties of Env describe the context in which epitopes are contained and are often indicative of important biological phenotypes, such as coreceptor tropism [75–78] or formation of some epitope groups [79, 80]. As expected [34, 81], the variable loops showed different patterns of historic changes in population diversity (see length of loops V1–V5 in Fig 1B and all feature types in S4 Fig). The V1 and V2 loops, which are located at the trimer apex and are mostly solvent-exposed [82–84], show increasing diversity in their lengths from Period1 to Period3. By contrast, the V3 loop, which is relatively cryptic on the Env trimer, shows little change over the past three decades. The V4 loop, which is solvent exposed but indispensable for trimer integrity and function [85], demonstrated a level of diversity similar to the V1 and V2 loops during Period1 but then diversified minimally over subsequent years. Therefore, although diversity of Env segmental features has generally increased from Period1 to Period3 (S5 Fig), different loops demonstrate different patterns of change.

In summary, sequence and antigenic features of Env show different patterns of change in the population over the past 30 y. We sought to examine the basis for such patterns, which could allow us to predict future changes in properties of circulating strains. For this purpose, we analyzed the spread of Env features from patient to population by studying the relationships between the following: (i) variance among strains cocirculating in the host at any time point, (ii) longitudinal divergence patterns in patients, and (iii) diversification of features in the population.

### For each feature of Env, the level of variance between strains cocirculating in the host is highly conserved among different individuals

**Fig 2:**
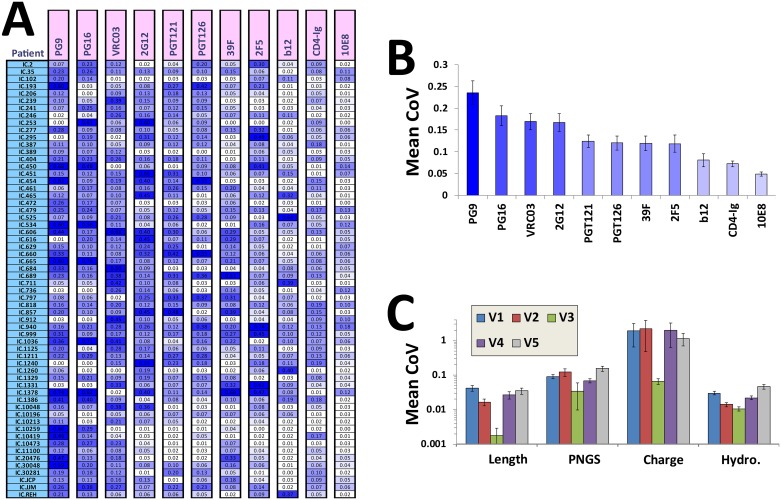
Antigenic and segmental features of Env show conserved levels of in-host variance. **(A)** Binding of the indicated probes was measured to Envs isolated from plasma samples of 60 HIV-infected individuals (2–8 Envs per sample). Values represent the variance in binding efficiency among Envs isolated from the same plasma sample, as calculated by the coefficient of variation (CoV). The CoVs are color-coded according to their values (darker shades of blue represent greater variance). **(B)** Mean CoVs of each feature for the 60 patients examined in panel A. Error bars represent the standard error of the mean (SEM). **(C)** The protein sequence of each Env was used to calculate the indicated features of the five variable loops of gp120, including amino acid length, mean hydropathy score, and the density of charge and potential N-linked glycosylation sites (PNGSs) (calculated as a fraction of the loop length). The CoV of features among Envs from the same plasma sample was calculated and averaged for all 60 patients. Data underlying this figure can be found in S6 Fig and S2 Data.

We first measured for each feature the level of variance among functional, cocirculating Envs. The coefficient of variation (CoV) of feature values among Envs isolated from the same plasma sample was calculated. Such measurements were performed for 60 cross-sectional samples. We found that some features demonstrate higher in-host variance than others (compare different columns in Fig 2A). For example, the CoV of PG9 was generally high in most patients (i.e., many hosts contained cocirculating viruses that had low and high binding efficiencies to PG9). For other features (e.g., 10E8 or b12), the variance among cocirculating strains was minimal (i.e., either high or low values in all Envs isolated from the same plasma sample). Consequently, the mean CoV values of each feature (averaged for all patients in each column) varied among probes (Fig 2B). The in-host variance pattern of each feature appeared to be conserved across different patients (see standard error bars in Fig 2B). Therefore, different structural features of Env appear to have different propensities for in-host variance.

We also examined the in-host variance for segmental features of the five variable loops of gp120. As expected, the V3 loop demonstrated relatively high conservation of length, charge, hydropathy score, and PNGS in each plasma sample (see mean CoVs in Fig 2C and the complete dataset in S6 Fig). The limited variation in the V3 loop likely reflects the restricted range of states this segment can assume and still maintain Env functionality [33, 86, 87]. Other variable loops, which show greater degrees of diversity in the population than V3 (Fig 1B and S4 Fig), also demonstrate higher in-host variance.

We emphasize that the above-described in-host CoV does not aim to quantify the absolute level of variance that may exist for each feature; such a value cannot be accurately approximated by the limited sampling we employ (2–8 Envs per sample). Instead, it serves as a relative measure of the propensity of features for variance in the infected individual at any given time point. Broad sampling (60 patients) allows us to identify such relative propensities with a good degree of confidence.

### The propensity for in-host variance (volatility) is a quantitative and conserved property of each feature

**Fig 3:**
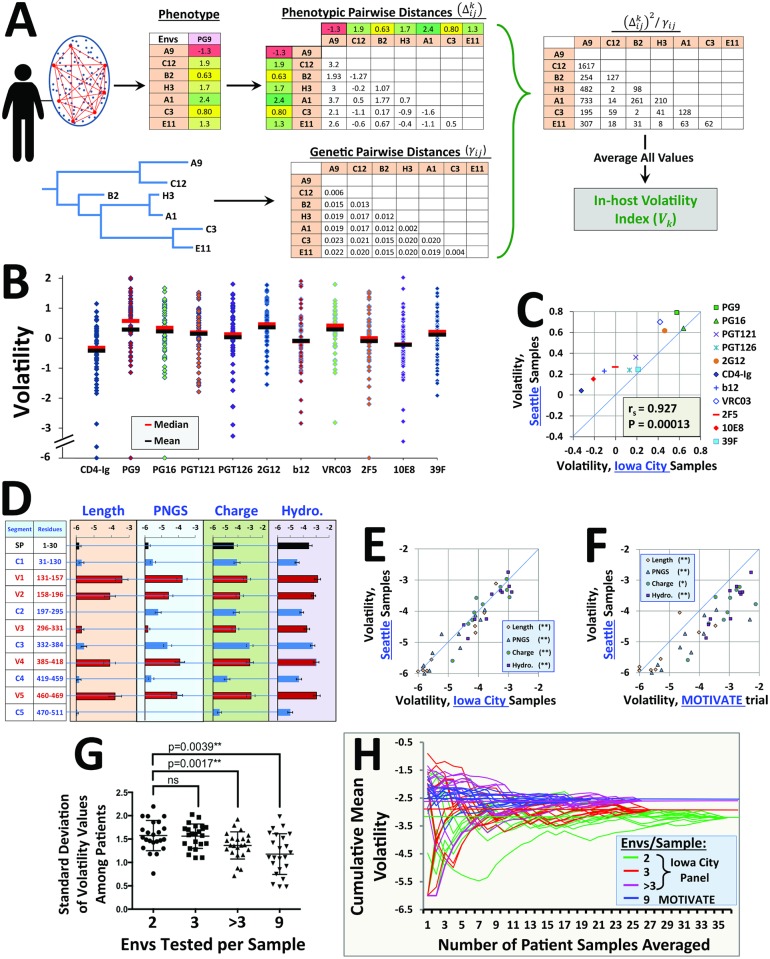
The volatility index is a conserved property of each feature. **(A)** Schematic of the approach used to measure the volatility index of a feature in a given plasma sample. The squared pairwise phenotypic distance between each Env pair in the plasma sample is calculated and divided by the genetic distance (based on amino acid sequence) that separates them. The ratio is averaged for all Env pairs in the sample to generate the feature volatility index for that plasma sample. **(B)** Volatility indices of antigenicity features measured in 60 patients. Calculated values were first log_10_-transformed. For averaging of the indices, all values smaller than –6 were assigned a value of –6. **(C)** Correlation between the median volatility index of antigenic features measured in 60 patients from Iowa City and 43 samples (from 15 patients) collected in Seattle. The ideal correlation (y = x) is shown by a blue line. **(D)** Mean volatility indices measured for segmental features using 60 samples from Iowa City. Amino acid positions of segments are numbered according to the HXBc2 convention [88]. Volatilities of all gp120 and gp41 segments are shown in S8A Fig. **(E, F)** Correlations between the mean volatility indices of segmental features measured using the above Env panels and a panel of Envs isolated from plasma samples of 20 patients collected for the MOTIVATE trial. Two-tailed *p*-values for the Spearman correlation test of each feature type are indicated (*, *p* ≤ 0.01; **, *p* ≤ 0.001). **(G)** Effect of Env sample size on differences between hosts in measured volatilities. We calculated the hydropathy volatility of the 23 segments of Env in Iowa City samples containing two, three, or more than three Envs and in MOTIVATE trial samples (average of nine Envs tested per sample). Each dot represents the standard deviation among patient volatilities for a given feature. Groups are compared using Wilcoxon signed-rank test. **(H)** The cumulative mean volatility of V1 loop hydropathy is shown for the above groups. For each group, ten random paths of calculation are shown, which represent different orders of cumulative averaging of volatility values. Error bars represent the SEM. Spearman rank correlation coefficient, r_S_; *p*-value, two-tailed test; ns, not statistically significant. Data underlying this figure can be found in S2 Data.

Many of the features that demonstrate increased in-host variance also show high levels of diversity in the population (e.g., epitopes of mAbs 2G12 and PG16). This suggested a possible association between in-host variance and the potential of features for diversification between hosts. We therefore sought to generate a more precise measure of the propensity of each feature for in-host variance, which could be used for quantitative comparison with its patterns of longitudinal divergence and population-level diversification. A small set of *env* genes randomly selected from circulating strains can show different genetic relationships; in some plasma samples, Envs differ by a single amino acid, whereas in other samples Envs can differ in ~10% of their amino acid content. To account for such differences, we corrected the phenotypic distance between Envs for the genetic distance that separates them (see schematic in Fig 3A). Pairwise phenotypic distances (e.g., differences in binding of a probe) between all Envs in a plasma sample were measured. Similarly, the genetic distances (based on amino acid sequence) between all Env pairs in a sample were determined (see Sequence analysis in the Materials and methods section and S7 Fig). The ratio between the squared phenotypic distance and genetic distance was calculated and averaged for all Env pairs in that plasma sample. This measure, which we designate the volatility index, describes the propensity of features for variance within the host (at a given time point) per genetic distance unit:

$$\;V_{k}=\frac{2}{n\left(n-1\right)}\Sigma_{i=1}^{n}\;\Sigma_{j=1}^{i-1}\;\frac{{\left(\Delta_{ij}^{k}\right)}^{2}}{\gamma_{ij}}\;\;\;\;\;\;\;\;\;k=1,2,\;\ldots ,\;\;m$$ (1)

where *V*_*k*_ is the volatility index of the *k*^th^ feature of Env, *m* is the total number of features, *n* is the number of Envs isolated from each plasma sample, $\Delta_{ij}^{k}$ is the difference between the values of the *k*^th^ feature for Envs *i* and *j*, and *γ*_*ij*_ is the genetic distance between amino acid sequences of Envs *i* and *j*. The volatility index is thus regarded as a constant property of each feature. It describes the propensity of the feature for variance per genetic distance unit (rather than the level of phenotypic variance). Accordingly, the few Envs we isolated with identical sequences (16 of 523) were disallowed. The volatility index of each feature was calculated in each of the 60 cross-sectional patients and log-transformed to reduce the effects of extreme values on averages (Fig 3B). We found that the mean volatilities of epitopes differed significantly; the epitopes of mAbs 10E8, 2F5, and b12 demonstrated low values relative to the epitopes of mAbs 2G12, PG9, and VRC03. A high correlation was observed between volatility indices measured in samples collected in Iowa City and Seattle (*p*-value of 0.00013 in a Spearman correlation test, Fig 3C).

We also examined the volatility indices of segmental features of gp120 (see Fig 3D and volatilities of all gp120 and gp41 segments in S8A Fig). Similar to the antigenicity features, volatility indices measured using plasma samples from Iowa City and Seattle correlated well (*p*-value < 10^−6^ in a Spearman correlation test, Fig 3E). Volatility indices were also measured using a third panel of plasma samples from 20 clade B–infected individuals who enrolled in the MOTIVATE trial, which examined efficacy of the CCR5 inhibitor Maraviroc [89, 90] (see alignment of these Envs in S4 Data). Only plasma samples collected prior to initiation of Maraviroc treatment were studied. The correlation between volatility indices measured in the Seattle and MOTIVATE panels was high (Fig 3F). Interestingly, the indices measured for the MOTIVATE samples were generally higher than those of the Seattle samples.

We hypothesized that the small differences in volatility between panels may result from differential sampling of Envs in each group; the average number of Envs isolated from each plasma sample in the Iowa City, Seattle, and MOTIVATE cohorts was 2.83, 3.04, and 9, respectively. We therefore compared the variance between volatility indices of features measured in Iowa City patients with two, three, or more than three Envs per plasma sample. As expected, the larger the number of Envs in each sample, the greater the similarity in volatility values among patients (see Wilcoxon signed-rank test comparing standard deviations of hydropathy volatilities among patients in each group, Fig 3G). Similar results were obtained for the antigenicity features, whereby variance in volatility values among patients was greater when only two Envs were isolated from a plasma sample relative to three or more than three Envs (*p*-values of 0.005 and 0.001, respectively). Therefore, the measured volatility index is not independent of sample size. Sampling of four to nine Envs per patient appears to generate a volatility value that is conserved among different individuals. The relationship between the number of patients studied and the cumulative mean volatility of V1 loop hydropathy is graphically demonstrated in Fig 3H. Greater sampling of Envs from each patient results in reduced variance and a mean volatility value closer to that of the MOTIVATE panel using samples from less patients.

In summary, the volatility index is a measure of the in-host propensity of features for variance at any given time point (rather than absolute variance in their values). This property of each feature is highly conserved in different patient populations, at least in the context of viruses from the same clade.

### The volatility index is associated with the level of feature divergence in longitudinally monitored patients

The conserved nature of volatility indices in different patients suggested that they may translate into defined longitudinal divergence patterns. We thus compared volatility with the mean divergence of Env feature values in a group of longitudinally monitored patients (see primary data and sequences in S1 and S3 Data). The panel includes 18 patients; for each, we examined two to five plasma samples separated by up to 11-y intervals (two to seven Envs were isolated from each sample). We note that 15 of the 18 patients were chronically infected at the time of first plasma sample collection. Only 3 patients (UW.1313, UW.1842, and UW.1406) may have been in the acute phase at the time of first sample collection (34, 74, and 159 d from first HIV+ test, respectively). Therefore, we view these analyses as generally representative of transitions between chronic states.

**Fig 4:**
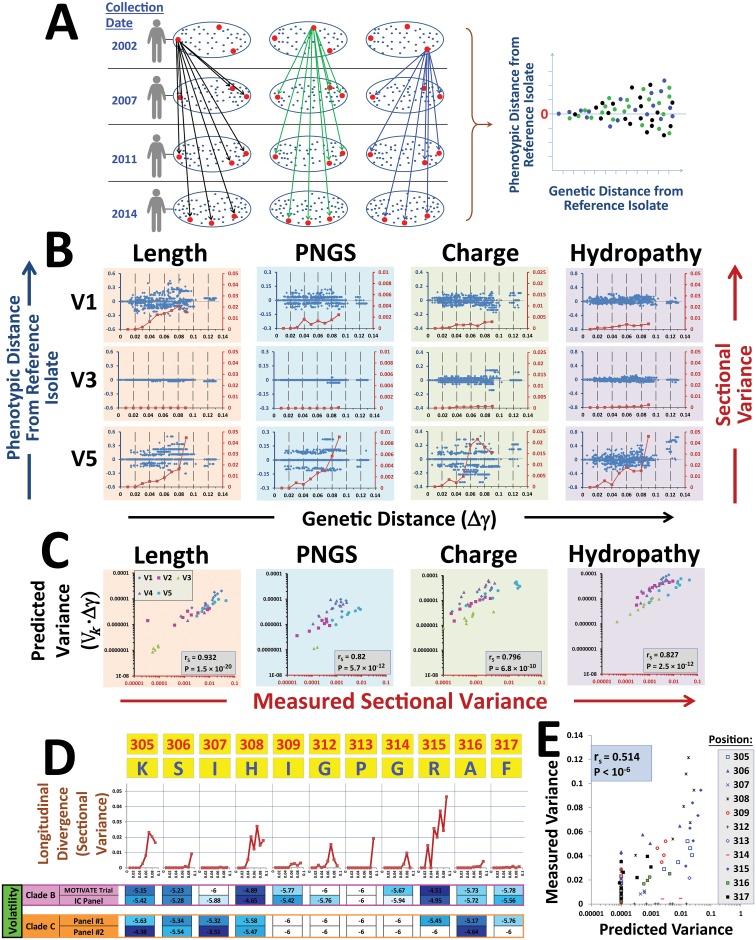
Relationship between the volatility index and longitudinal divergence of Env features. **(A)** Approach used to measure diversification of phenotypes between mixed states. Phenotypic and genetic distances were measured between each reference Env from the first plasma sample and all other Envs. **(B)** Longitudinal divergence of segmental features measured for loops V1, V3, and V5 in 18 patients monitored for up to 11 y. Data represent the phenotypic pairwise distances between each reference isolate and all other Envs from that patient and are divided by the value of the reference isolate. All pairwise distances measured for all patients are shown. To monitor the progression of variance and allow equal representation for all patients, we divided the *x*-axis into sections of 0.01 genetic distance units (see vertical lines). For each section, all phenotypic pairwise distances from the same patient were averaged. Variance among patient averages for the same section was then calculated (labeled by red squares). Because of the small number of isolates in sections that describe larger genetic distances, calculations were performed only for sections of 0.01 to 0.09 distance units. Data describing divergence of all loops are shown in S9A Fig. **(C)** Correlation between the measured variance of features in each section and the predicted variance (calculated as the product of the volatility index and the genetic distance units of the section). **(D)** Longitudinal divergence of hydropathy score of the indicated V3 loop residues, as quantified by the sectional variance value. The hydropathy volatility at each position for panels of Envs from clades B and C is shown. **(E)** Correlation between predicted and measured hydropathy score for V3 loop crown residues at genetic distances of 0.01 to 0.09 units. Data underlying this figure can be found in S2 Data.

To measure longitudinal feature divergence from the mixed state that exists in these patients, we used each of the isolates from the (chronologically) first plasma sample as a reference (designated herein as the reference Env(s)). Phenotypic and genetic distances between each reference and all other Envs were then measured (see schematic in Fig 4A). Variable loop features demonstrated gradual divergence with increasing genetic distance from the reference Env(s) (see loops V1, V3, and V5 in Fig 4B and all loops in S9A Fig). As genetic distance increased, features diverged to different extents. The V1 and V5 loops exhibited the greatest degree of divergence for most feature types. By contrast, the V3 loop maintained conserved feature values, likely reflecting the limited range of lengths, PNGS, and charge values that allow effective Env trimer packing and interaction with a coreceptor on target cells [86, 87]. To quantify the patient-averaged divergence of features, we dissected the range of genetic distances into sections of 0.01 units (see vertical dashed lines in Fig 4B) and examined the progression of variance. For some features, the trend of gradual divergence from the initial state is disrupted at genetic distances greater than 0.08, likely because of the relative paucity of in-host Env pairs with such genetic distance separation. These highly divergent Envs are mainly derived from two patients that contain major recombination events (UW.1140 and UW.1393).

The feature-specific patterns of divergence suggested that we may apply the volatility indices (measured using the cross-sectional patient samples) to predict the range of feature values that developed in the longitudinal patients. Based on the dispersion patterns of values (Fig 4B), we modeled phenotypic changes as a linear diffusion process that progresses with genetic distance (*γ*) from the reference state. As such, we expect it to satisfy the following stochastic differential equation:

$$dX_{k}\left(\gamma \right)=\mu_{k}d\gamma +\sigma_{k}dW\left(\gamma \right)$$ (2)

where *X*_*k*_ is the value of the *k*^th^ feature, *μ*_*k*_ is a constant and represents the mean rate of change (drift) parameter, *σ*_*k*_ represents the “diffusion” index of the feature (i.e., the tendency for dispersion of feature value), and *dW*(*γ*) describes the incremental contribution of a random variable that is normally distributed with mean 0 and variance *d*(*γ*). Traditionally, contribution of the stochastic component (i.e., random variable) to evolution of features is described by a Wiener process denoted by *W(t*) [91]. To describe evolution of Env features, changes are indexed by genetic distance (*γ*) rather than time (*t*). At this stage, we assume the absence of a deterministic component (*μ*_*k*_) to the changes in feature value (i.e., the absence of a constant phenotypic drift). Thus, for a process that is solely driven by the stochastic component, variance of the increments to feature *X*_*k*_ is described by:

$$\text{Var}\left(\Delta X_{k}\right)=\sigma_{k}{}^{2}\cdot \Delta \gamma$$ (3)

where Δ*γ* represents the genetic distance between the reference and tested isolates. We examined whether we can substitute the volatility index measured using the cross-sectional samples (Eq 1) for *σ*_*k*_^2^ to predict the variance that developed in the group of longitudinally monitored patients at each genetic distance section from the (mixed) reference state. The measured sectional variance value was compared with the predicted value, calculated as the product of the volatility index and the genetic distance of the section analyzed. Strong relationships were observed between predicted and measured variance for the length and hydropathy of the five variable loops (Fig 4C). Predictions of charge and PNGS divergence were also good, although some differences were observed between variable loops in the relationships between volatility and divergence (compare with uniform slopes of length and hydropathy). Therefore, translation of volatility into longitudinal divergence is relatively similar for some feature types whereas other features exhibit more complex patterns of translation.

As an alternative to the sectional approach, we also analyzed the overall pattern of divergence in each patient (fit to a single linear regression model), followed by averaging of all patients. Divergence of each feature calculated using a simple regression model correlated well with the volatility index (S9B Fig). By applying a sectional approach, we minimize the effects of changes that occur at greater genetic distances on the overall divergence pattern in each patient (e.g., by recombination events) and can thus analyze the progression of divergence more accurately.

We also examined the longitudinal divergence of individual amino acid positions of Env and the association of this process with their volatility indices. The V3 loop of HIV-1 often evolves during the course of infection and allows a switch from utilization of the CCR5 coreceptor to CXCR4 [33, 35]. Longitudinal analyses of V3 loop features show it is highly conserved in length and PNGS whereas charge and hydropathy can alter over the course of infection (Fig 4B). We examined the longitudinal evolution of hydropathy of each amino acid position of the V3 loop crown. Different propensities for longitudinal divergence were observed, as measured by the sectional variance values (Fig 4D). We also measured the hydropathy volatility of each amino acid position by the same approach applied to measure segmental hydropathy volatilities, using Env panels from Iowa City and the MOTIVATE trial. Since it was previously suggested that exposure of V3 loop crown residues differs in clades B and C viruses [73], we also measured volatility in two independent panels of Envs from plasma of clade C–infected individuals [92, 93] (S5 Data). Indeed, notable differences were observed between volatility of V3 loop tip residues in the panels of Envs from clades B and C, specifically for the sequence His-Ile-Gly-Pro-Gly-Arg at positions 308–315 (Fig 4D). Volatility in this region was higher for clade B Envs. Interestingly, the same positions also exhibit increased Shannon entropy values in clade B relative to clade C viruses [73]. A strong relationship was observed between the measured sectional divergence of each amino acid hydropathy and the predicted divergence (calculated by the product of hydropathy volatility and sectional genetic distance, Fig 4E).

### In-host divergence of antigenic features and associated asymmetry of increments

**Fig 5:**
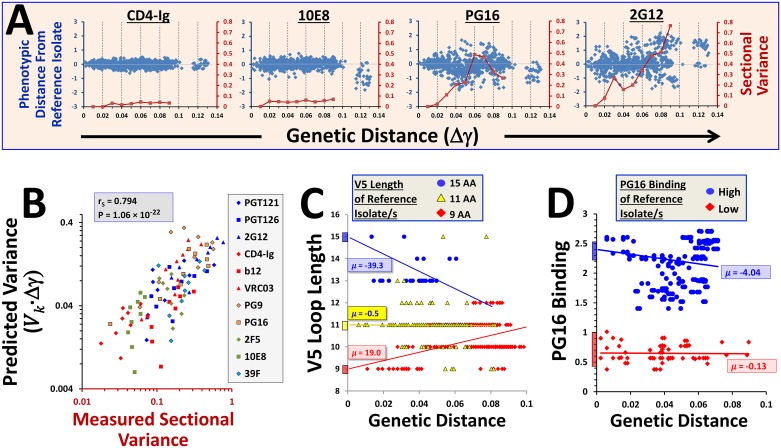
Longitudinal divergence of Env features and associated asymmetry of increments. **(A)** Evolution of variance in 18 longitudinally monitored patients. Divergence of all 11 features is shown in S10 Fig. **(B)** Correlation between predicted and measured antigenic variance that developed at each genetic distance section (in the range of 0.01 to 0.09 units). **(C)** Changes in length of the V5 loop in longitudinally monitored patients with increasing genetic distance from the reference isolate. Evolution was examined separately for patients in which the V5 loop of the reference Env(s) was short (9 amino acids, red), intermediate (11 amino acids, yellow), or long (15 amino acids, blue). A least-squares regression line was fit to each dataset, which describes the mean change in feature value per genetic distance unit; the slope of the line (*μ*) is indicated. **(D)** Changes in binding efficiency of mAb PG16 in longitudinally monitored patients from different reference states. Data are colored according to the value of their reference state. The vertical colored bars by the *y*-axis represent the range of values of the reference isolates. Data underlying this figure can be found in S2 Data.

Divergence of antigenicity features was examined in the longitudinal patients. In accordance with their patterns of population diversity (Fig 1A) and in-host variance (Fig 2B), the epitopes of mAbs 2G12 and PG9 showed significant propensities for longitudinal divergence (see Fig 5A and entire dataset in S10 Fig). By contrast, divergence of the CD4-binding site probes b12 and CD4-Ig and the MPER-targeting probes 10E8 and 2F5 was limited. A strong relationship was observed between the predicted and measured sectional divergence values (Fig 5B). In some cases, (e.g., PG9 and 2F5), the gradual divergence from the initial state becomes less apparent at genetic distances greater than 0.08. We attribute this change in pattern to the relative paucity of Env pairs with such large genetic distance separation. Nevertheless, overall the selective forces that act in the individual appear to be sufficiently stable over time to allow application of the volatility index to predict the propensity for divergence of each feature.

The lengths of the Env variable loops are not normally distributed in the population (see histograms and results of D'Agostino–Pearson Omnibus test in S11 Fig). The observed patterns suggest existence of constraints that may limit the appearance of Envs with loop lengths below or above certain values. Such constraints may affect the longitudinal divergence from some reference states. For example, analysis of changes in the length of V5 from a reference state that contains only 9 amino acids show mainly positive increments relative to the more symmetric changes from a reference state with 11 amino acids (Fig 5C). Thus, restraints imposed by biological properties of Env can affect feature evolution. However, such bounds are often not absolute. For example, a V5 loop that contains 15 amino acids is less preferred; longitudinal analyses reveal that such Envs will normally “drift” back to a state that contains a shorter V5 loop (Fig 5C). Nevertheless, two patients in the Iowa City panel have viruses that contain even larger (17 amino acid) V5 loops. Therefore, the restraints imposed on V5 length are better described by preferences for specific states. Restraints can also be applied by inherent properties of the features themselves. For example, acquisition of an epitope is (in many cases) less likely than loss of the epitope (e.g., by a single point mutation). Diffusion from a state that lacks an epitope is thus restrained (Fig 5D). Therefore, the short-range diffusion of feature values (i.e., for small genetic distance increments) is controlled by the initial state of the feature and by the volatility index. Accordingly, rather than treating evolution as a symmetric diffusion process that is only determined by volatility, we can introduce a state-specific drift component (*μ*_*k*_ (*X*_*k*_ (*γ*))), which is composed of the restraining forces imposed by properties of the molecule or the feature. For a process affected by volatility and such a drift the incremental change in feature value can be described by:

$$dX_{k}\left(\gamma \right)=\mu_{k}\left(X_{k}\left(\gamma \right)\right)d\gamma +\sqrt{V_{k}\cdot d\gamma }\;dW\left(\gamma \right)$$ (4)

We assume that over short genetic distances, the drift component is a constant that depends on the reference state. For longer-range paths, the expression should accommodate the dynamic changes that occur over increasing genetic distances (see Discussion section).

In summary, the volatility index of each Env feature provides an accurate measure of the mean degree of longitudinal divergence expected to occur in patients. Although different pressures are likely applied in different patients, the “noise” measured in the individual at any moment is highly correlated with the propensity for change over the course of time. A few features demonstrate imperfect correlations between the predicted and measured divergence (e.g., PG9, 10E8, and 2F5). Such variations may result from the following: (i) limited sampling of some genetic distance sections (i.e., paucity of Env pairs at greater genetic distances), (ii) different pressures applied in different individuals, (iii) low volatility and divergence values (e.g., for mAbs 10E8 and 2F5), and (iv) lack of complete diffusion symmetry from all states (i.e., dependence on initial feature values). Nevertheless, overall the volatilities of individual amino acids, Env segments, and epitopes are highly conserved among patients and allow accurate predictions of the mean divergence expected in a group of longitudinally monitored individuals (Figs 4C, 4E and 5B).

### In-host volatility predicts the historic diversification patterns of Env features in the population

**Fig 6:**
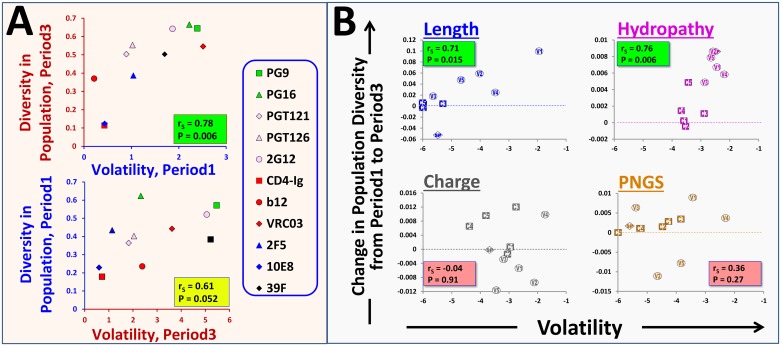
Relationship between in-host volatility and population-level diversity of Env features. **(A)** Volatilities measured in samples collected in Iowa City during Period1 or Period3 are compared with the diversity of each feature (calculated by the standard deviation of the feature value) in Iowa City during Period3 or Period1, respectively. **(B)** Comparison between in-host volatility and diversification of gp120 features between Period1 and Period3 in Iowa City. Volatility was calculated using the 20 samples of the MOTIVATE trial. SP, signal peptide; V, variable loop; C, constant region. Comparison between volatility and diversity of features in Iowa City during Period3 is shown in S12 Fig. Data underlying this figure can be found in S2 Data.

Diversification of Env features in the population is affected by their propensity for divergence within patients and the selective forces that act during transmission [49, 94, 95]. We hypothesized that if longitudinal patterns of change are sufficiently conserved among different individuals and if selective pressures that act during virus transmission have comparable magnitude, then volatility could be applied to predict population-level changes in each feature. We thus examined whether the diversification patterns of Env features over the past three decades (Fig 1) can be explained by differences in feature volatilities. We tested this relationship for the antigenicity features; volatility was measured using patient samples from Period1 or Period3 and compared with the diversity of the features in the population during Period3 or Period1, respectively. A linear relationship between volatility and diversity was observed (Fig 6A). Interestingly, volatility in Period1 samples served as a better predictor of feature diversity during Period3 than vice versa. Such a pattern could result from limited diversity of these features during Period1 or from changes in volatility from Period1 to Period3 (see S5, S8B and S8C Figs). Nevertheless, whether in-host volatility is stable or dynamic over time, this inherent property of each feature is translated in a defined and predictable manner into its population-level diversity.

We also examined the relationship between volatility and historic changes in segmental features of gp120. Volatility measured using the 20 patient samples of the MOTIVATE trial was compared with the historic diversification of each feature in Iowa City (measured by the difference in diversity between Period3 and Period1). For both segmental length and hydropathy, we observed a clear linear relationship between in-host volatility and the changes that occurred in feature diversity between the two periods (Fig 6B). By contrast, charge and PNGS showed a nonuniform association pattern. Comparison between volatility and P3 diversity of charge and PNGS also showed that comparable levels of population diversity may exist despite significant differences in volatility (S12 Fig).

Therefore, all antigenic features we tested and some segmental feature types show direct “translation” of their in-host volatility into population-level diversity. For other feature types, translation is not identical for all segments, suggesting potential involvement of additional factors. The above-described changes in segmental features describe evolution of Env structure at “low resolution” (i.e., the context in which epitopes are expressed). We sought to examine the ability of the diffusion-based model to predict changes in amino acid sequence of antigenically significant regions of Env.

### Predictions of population-level changes in amino acid sequence of the V3 loop crown and MPER

**Fig 7:**
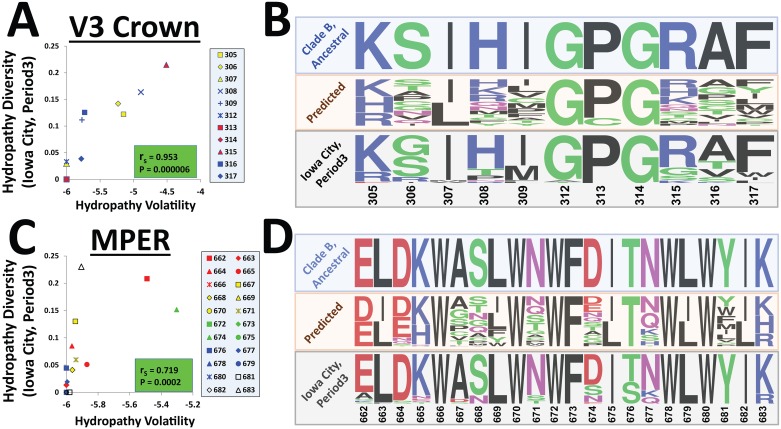
Prediction of population-level changes in sequence of the V3 loop crown and membrane-proximal ectodomain region (MPER). **(A)** Hydropathy volatility of residues 305–317 was calculated using the 20 patient samples of the MOTIVATE trial. Values are compared with diversity of the hydropathy score of each position in Iowa City during Period3. **(B)** To predict the amino acid variants that appeared in the population, we measured for each position the volatility of charge, molecular weight, and hydropathy. The likelihood of changes from the consensus sequence in Iowa City during Period1 (also the clade B ancestor) to each possible amino acid variant was then calculated using a joint probability density function, which combines the likelihoods of the transition for the three feature types (Eqs 5 and 6). A schematic describing the approach is provided in S13 Fig. Sequence logos represent the ancestral strain, the predicted variants (calculated using the joint probability density function), and the amino acid variants at each position that circulated in the Iowa City population during Period3. Calculations were solely based on the volatility of each feature; substitution likelihoods of amino acids were not taken into consideration. **(C)** Relationship between volatility and diversity of the hydropathy score of MPER residues in Iowa City during Period3. **(D)** Prediction of the MPER amino acid variants that evolved in the Iowa City population using the joint probability density function. Data underlying this figure can be found in S2 Data.

We examined whether application of the volatilities of individual positions of Env would allow us to predict the specific amino acid variants that appeared in the Iowa City population over the course of three decades. We first examined the V3 loop crown. Similar to the segmental features, hydropathy volatility of each amino acid (calculated using the 20 plasma samples of the MOTIVATE trial) correlated well with its diversity in the Iowa City population during Period3 (Fig 7A). Positions that show limited or no variance in the infected individual at any time point also show minimal longitudinal changes (Fig 4D) and were unaltered in the population over the course of three decades.

We hypothesized that if the sequence of Env was sufficiently conserved during Period1 then we could predict both the level of diversity that developed and potentially the specific amino acid variants that appeared in the population. For this purpose, we applied the volatility of three features of each amino acid (charge, molecular weight, and hydropathy) in a joint probability density function. The consensus of Period1 Envs in Iowa City was used as the reference (“ancestral”) sequence. For each position, we then calculated the likelihood of each amino acid variant (*k*) to evolve from the ancestral state (*α*) based on the propensity for change in that feature (i.e., volatility). We thus treat the changes that occur in properties of amino acids at each position as a one-dimensional random walk; for each position, we measure the likelihood of the feature value to “diffuse” away from its ancestral state to any other amino acid value. A schematic example of the approach is provided in S13 Fig. We assume that the above feature types are normally distributed and apply a probability density function:

$$\begin{array}{l} P_{k}^{i}=f(x_{k}|\alpha_{k},V_{k})=\frac{1}{\sqrt{2\pi V_{k}}}e^{\frac{-{\left(x_{k}^{i}-\alpha_{k}\right)}^{2}}{2V_{k}}}\\ k=1,2,\;\ldots ,\;m;\;i=1,2,\;\ldots ,\;n \end{array}$$ (5)

where $P_{k}^{i}$ is the likelihood of obtaining the *i*^th^ variant of an amino acid based on volatility of the *k*^th^ feature type (*V*_*k*_), $x_{k}^{i}$ is the value of the *i*^th^ variant of feature *k*, and *α*_*k*_ is the value of the reference state for feature *k*. This calculation is repeated for each feature type (hydropathy, molecular weight, and charge), and the combined likelihood ($P_{Total}^{i}$) of obtaining the *i*^th^ variant of an amino acid based on volatilities of all feature types is defined by:

$$P_{Total}^{i}=\prod_{k=1}^{m}\;P_{k}^{i}$$ (6)

We compared the consensus sequence of the V3 loop crown in Iowa City during Period1 (similar to that of the clade B ancestor) with the distribution of sequence variants in the Iowa City population during Period3 and the predicted range of variants (see sequence logo representation in Fig 7B). We found that the expression performed well; positions that were predicted to diversify minimally (based on volatility of the three features) also showed limited or no diversity in the population. Positions that showed high volatility in patients also demonstrated greater diversity in the population. Thus, using measurements from 20 plasma samples (and the ancestral sequence) we can predict for many positions the diversity that developed over the course of 30 y. We emphasize that the approach does not take into account the likelihood of each amino acid substitution; incorporation of substitution likelihoods further improves the predictive capacity of this basic model. We also note that the model treats all amino acid residues as independent variables and does not acknowledge the well-defined networks of association that exist within the V3 loop [89, 96, 97]. Inclusion of such considerations is expected to improve performance of the model still further.

The model was also tested for the ability to predict changes in the MPER of gp41. Several epitopes of broadly neutralizing antibodies map to this Env region [68, 98, 99]. Similar to the V3 loop crown, hydropathy volatility of each amino acid position correlated well with its diversity in the population during Period3 (Fig 7C). The N-terminal portion of the MPER, which contains the 2F5 epitope, was relatively more volatile than the C-terminal part, which contains the 10E8 epitope. Such a pattern is expected of the conserved C-terminus, which interacts with cholesterol [100–102] and can regulate global sensitivity of Env to antibodies [103]. These data also correlate well with antigenicity results, which show conserved integrity of the 10E8 epitopes but some diversification (albeit limited) of the 2F5 epitope over the past three decades (Fig 1A).

Interestingly, the highest volatilities in the MPER were measured at positions 662 and 674. Position 662 is associated with changes that regulate coreceptor tropism, from CCR5 to CXCR4 [104]. Position 674 is associated with regulating the global responsiveness of Env to inhibitory agents (such as antibodies) and to Env-activating molecules (such as the coreceptors) [59]. Changes at this position allow transition from a state of increased fusogenicity and sensitivity to antibodies (advantageous in vitro) to a state of reduced fusogenicity but also reduced sensitivity to antibodies (advantageous in vivo) [59, 105, 106]. The relatively frequent “fluctuations” at these positions may allow the virus to achieve such phenotypic switches more effectively and thus to rapidly adapt to the environment.

An interesting discrepancy was observed for position 681 between the intermediate-level volatility measured in the MOTIVATE trial samples and the complete conservation of this residue (Tyr) in the Iowa City population and among all group M, N, O, and P strains of HIV-1 [107]. Indeed, Envs containing mutations at this position are often fusion-competent [108]. That variants at position 681 are found among cocirculating strains (in highly sampled individuals) but do not appear in the general population suggests the involvement of selective pressures applied on this site over time in the individual or during virus transmission. Discrepancies between the levels of in-host variance, longitudinal divergence, and population diversity allow us to identify bottlenecks that restrict the continuity of heterogeneity across time and different patients and permit preferential spread of only selected forms of the virus.

Similar to the V3 loop crown, application of the joint probability density function allowed us to predict well the positions that remained unchanged and often the variants that evolved from the ancestral state and currently circulate in the population (Fig 7D). Therefore, through limited sampling of the population (i.e., using volatility indices measured from 20 patient samples) we can predict the diversity that developed at many Env positions and approximate well the nature of the specific amino acids.

## Discussion

**Fig 8:**
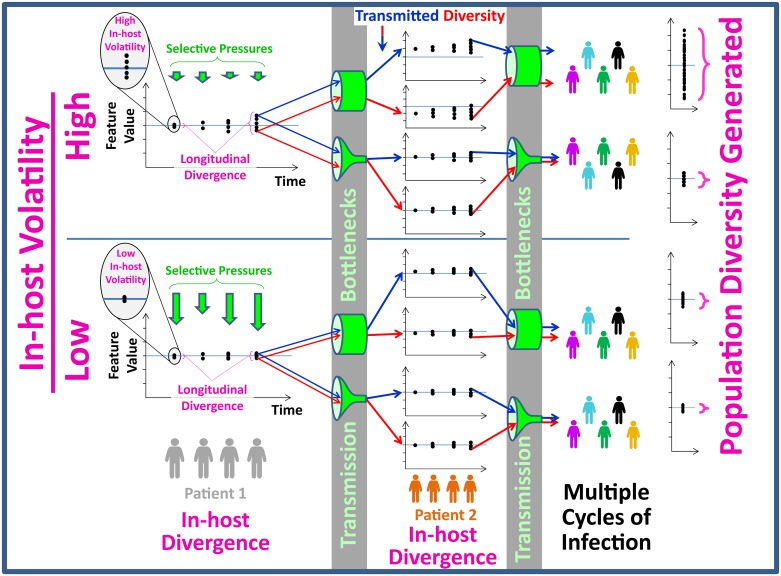
Spread of HIV-1 Env features from patient to population. Evolution of viruses circulating in the host is controlled by immune and fitness pressures. The collective effects of such pressures determine the permissiveness for variance of each feature at any given time point (i.e., volatility). The propensity for longitudinal divergence of features is closely related to volatility; small degrees of variance can be amplified over time to increase the range of values. During transmission of the virus between hosts, some features are subjected to selective pressures specific to the transmission process (bottlenecks), which limit potential diversity in the recipients. Occurrence of these processes across multiple patients and transmission events defines the range of feature values in the population. Thus, measured across three decades, the in-host volatility and transmission bottlenecks dictate distribution of each feature in the population.

More than three decades after identification of HIV-1 as the causative agent of the AIDS pandemic, the road to an effective vaccine still appears to be long and winding [109]. A primary challenge we face in immunogen design is the tremendous diversity of Env variants circulating in the population and the continuing diversification process of this protein [54, 110]. Our data indeed show that many Env features (sequence and structural) were relatively conserved among isolates in the 1980s. From this “originator” state, different features diversified to different extents. To identify clues that could help predict the patterns of spread, we compared for each feature its propensities for in-host variance, longitudinal divergence, and population-level diversification. Our results are summarized by the schematic in Fig 8. The level of in-host “noise” (i.e., volatility) describes the propensity of features for variance in patients at any given time point; it is determined by the selective pressures applied (i.e., the requirement of Env to maintain function and resistance to immune pressure). Volatility guides the longitudinal divergence of features in the infected individual. Translation of this pattern across patients is controlled by transmission bottlenecks, which allow preferential spread of some forms between individuals [95, 111–116]. Therefore, the driving forces behind phenotypic diversity and its translation across time and different patients are measurable. Given the conserved nature and dominant contribution of volatility to this process, we can apply it to approximate the distribution of feature values in the population, estimate expected future changes, and potentially identify phenotypes selected during transmission.

### Evolution of HIV-1 Env features as a diffusion process

For all Env features, variance gradually increases with genetic distance from the initial reference state(s), both within the infected individual and in the population. Different features demonstrate different (but conserved) progression “rates” of variance per genetic distance unit. Such propensities for variance can be clade-specific and potentially account for observed diversification patterns of features in the different clades. We thus model the evolution of feature values as a linear diffusion process that is controlled by volatility. Accordingly, the value of feature *X*_*k*_ at a given genetic distance *γ* from the reference state *X*_*k*_(0) can be described by:

$$X_{k}\left(\gamma \right)=X_{k}\left(0\right)+\mu_{k}(X_{k}\left(\gamma \right))\cdot \gamma +\;\sqrt{V_{k}\cdot \gamma }\cdot W\left(\gamma \right)$$ (7)

Therefore, *X*_*k*_(*γ*) is normally distributed, with an expected value (mean) of *X*_*k*_ (0) + *μ*_*k*_ (*X*_*k*_ (*γ*)) · *γ* and variance *V*_*k*_ · *γ*. The expression distinguishes between contributions of the stochastic and deterministic components. For diffusion across short genetic distances, we can assume that the drift component *μ*_*k*_ (*X*_*k*_ (*γ*)) is still controlled by the reference state value. However, at greater genetic distances from the reference state, the drift is less well defined. In this work, which primarily focuses on the population-level spread of features, we acknowledge the presence of a state-specific deterministic drift but primarily focus on the stochastic component. By presenting our results in the context of the complete model, we aim to demonstrate how evolution of phenotypes can be captured by a diffusion process and the possibilities this approach offers.

Changes in antigenicity features of Env (in patients and population) are treated as a diffusion process—a Markov process indexed by genetic distance (rather than time) with sample paths that are almost surely continuous. Sample paths for binding efficiencies of probes are indeed clearly continuous. We apply a similar approach for analyzing the chemical properties of amino acids (charge, hydropathy, and molecular weight), which are approximated here to be normally distributed. Accordingly, the process of changes in chemical properties of amino acids from a defined reference state represents a diffusion approximation.

Evolution of Env features differs from other systems typically described by random walk-like changes (e.g., displacement of colloidal particles or evolution of stock option prices) in several ways: (i) progression is indexed by genetic distance, (ii) features are characterized by partial sampling from a mixed state (characterization of all states present is not viewed as a feasible option), and (iii) increments cannot be assumed to be completely independent (although they are treated as such in this study). Specific characteristics of this form of feature evolution, which we expect can be applied to describe other biological systems, are discussed below.

### Defining the stochastic process *W*_γ_ as a generator of random variables

Stochastic differential equations are used in many scientific disciplines to model systems that contain a dominant “uncertainty” component [117–124]. In many of these studies, a generalized Wiener process is used to describe the random function. It is a normally distributed, continuous-time stochastic process with independent increments. Here, we define a stochastic process we designate **W**_**γ**_ as the single “generator” of random variables (i.e., as the randomness-introducing function). Its contribution is controlled by the volatility of the feature. Since we preselect all Envs for functionality, the increments represent the combined effect of diversity-increasing forces (mutations and recombination events) and diversity-decreasing forces (immune- and fitness-selective forces applied on the molecule). Therefore, **W**_**γ**_ is likely better represented not by a single continuous process. Indeed, two types of events introduce genetic (and thus phenotypic) change; point mutations and recombinations [8, 10, 11]. In 2 of the 18 longitudinal patients, we observed major recombination events (as predicted by the RDP4 software [125]). Both patients showed significant genetic distance leaps (see extreme genetic diversities in Figs 4B and 5A and heavy-tailed distribution in S14 Fig). Such genetic “leaps” can potentially account for “leaps” in virus phenotypes. Therefore, although we currently model increments as part of a single, normally distributed process that we designate **W**_**γ**_, the data could be represented more accurately by several random processes that introduce different increments, such as those described by the Merton jump-diffusion model of option pricing [126, 127]. Future studies will compare the effects of single-site mutations and recombination events on evolution of feature values.

### The feature volatility index

The volatility index describes the propensity of each feature for variance with increasing genetic distance. Conservation of volatility across multiple patients from different geographic regions illustrates its robustness in the context of within-clade analyses. Whereas volatility is treated in this study as a constant, it is clear that, similar to the drift component, this parameter may show state-conditional effects and potentially non-Markov properties [128, 129].

Volatility can be divided into two “tiers.” Preselection volatility describes the range of feature values that can appear in the infected individual in the absence of any restraining selective pressures. This theoretical range of variants includes all potential progeny isolates, functional and nonfunctional. The measured (postselection) volatility accounts for the effect of immune and fitness constraints and describes only the functional variants that circulate in patients. Assuming a constant rate of mutations and a homogenous distribution of the mutations across the *env* gene, preselection volatility is controlled only by the “complexity” of the feature. Complexity describes sensitivity of the feature value to a random change in amino acid sequence (i.e., the number of residues that affect the feature). In our analyses, we rendered complexity comparable for segmental features of the variable loops by normalizing each for loop length (i.e., volatility is calculated per amino acid). Therefore, differences between measured volatilities of segmental features reflect the effects of selective forces rather than feature complexity. By contrast, for the antigenicity features, different epitopes involve different numbers of residues in formation or maintenance of their integrity (i.e., varying degrees of complexity). Whereas most Env epitopes recognized by neutralizing antibodies are discontinuous, we included two probes that recognize linear epitopes; 10E8 and 2F5. Their epitopes thus likely have relatively low complexity. Indeed, the volatility of both epitopes was low. In particular, volatility of 10E8 was also low since it is associated with regulating function and global antibody sensitivity of Env [103, 130] and is therefore under greater selective pressure. In a similar manner, volatility of individual amino acids is controlled by immune and fitness pressures applied on each position. The effect of such pressures is associated with the degree of solvent exposure of the residue [131, 132]. As expected, our data suggest that a cryptic state is associated with lower volatility (Fig 4D).

### Modeling evolution by partial sampling from mixed states

Our analysis of phenotypically mixed states through partial sampling does not seek to capture the entire diversity in the system; it is appreciated that many states remain unsampled. Envs that we isolated and tested likely represent the more abundant quasispecies circulating in the individual and thus (potentially) are more transmissible relative to other variants and quiescent forms [36, 49, 95, 133]. The basic model described here does not (yet) account for the potentially dynamic nature of the selective forces applied; all Envs from the longitudinal patients are treated as part of one compartment and indexed by genetic diversity from the reference isolate(s). Accordingly, the factors that can alter with time in the infected individual (e.g., replication rate and selective pressures) are treated as uniform for all samples. Similarly, other than excluding all patients treated by entry inhibitors, the model does not yet account for the potential effects of antiretroviral therapy on virus diversity at each time point. For increased accuracy, the models will be adapted to describe the state of such dynamic variables of the virus and immune system in each unique environment as well as functional features of Env [114, 115].

### Application of the model for HIV-1 Env immunogen design

Vaccine immunogens are selected according to the clades that circulate in the target population. Our results suggest that in addition to phylogenetic considerations, vaccines should likely also address the dynamic nature of Env structure in the population. For example, the epitopes of mAbs VRC03 and 2F5 were similarly distributed in HIV-infected individuals during the 1980s (Fig 1A). However, the VRC03 epitope is currently present only in ~30% of the population (and will likely continue to disappear) whereas the 2F5 epitope is found in ~90% of circulating strains. When such historic information is available (by extensive sampling of infected individuals over the course of decades), predictions of future states can be generated based on past patterns of change. However, such information is not available for the many HIV-1 clades and recombinant forms that infect individuals worldwide. Therefore, reliable predictors of changes expected to occur in Env structure are required. The volatility index provides the clues necessary to achieve this goal, based on minimal sampling of patients. Knowledge of the population longevity of epitopes will likely be beneficial to immunogen design. The basic tools described here allow accurate predictions of changes in feature diversity and (for some amino acid positions) the nature of variants expected to evolve at key antigenic sites. By taking into account the network of associations that exists within Env and the coevolutionary patterns of its components [96, 97], the accuracy of these predictions can be further improved. The flexibility imparted by the model, which is based on the stochastic component of change but also combines the effects of deterministic drifts, will likely facilitate analyses of changes in other dynamic biological systems.

## Materials and methods

### Ethics statement

This study involved the use of peripheral blood samples from HIV-infected adult subjects who gave informed consent under clinical protocols approved by the participating institutions' human use review boards, including those at the University of Washington at Seattle Center for AIDS Research (CFAR) and University of Iowa (IRB numbers 8807313, 200010008, and 2010101730). This study did not involve animal research (vertebrate animals, embryos, or tissues). This is not a field of study, nor did it involve collection of plant, animal, or other materials from a natural setting.

### RNA extraction, cDNA synthesis, and single genome amplification of HIV-1 *env* genes

Blood was collected from patients in tubes containing acid citrate dextrose. Plasma was then separated, divided into aliquots and stored at −80°C until use. Viral load in all plasma samples was measured; samples that contained a measurable viral load (generally greater than 1,000 copies per ml) were further processed for isolation of the viral *env* genes, as described below. To isolate RNA, plasma samples were centrifuged twice at 5,500 *g* for 5 min to remove cell debris. Virus particles were then pelleted by centrifugation at 21,000 *g* for 3 h at 10°C, and the supernatant was removed and stored at –80°C for future use. Pellets were then resuspended in 140 μl of 150 mM NaCl, and RNA was extracted using QIAamp MinElute Virus Spin kit (Qiagen). Extraction was performed as recommended by the manufacturer except that instead of using carrier RNA, the AL buffer was supplemented with 40 μg/ml acrylamide (Sigma) to allow RNA sequencing of the samples. RNA was recovered from spin columns in 30 μl water and then either frozen at −80°C or immediately used to synthesize cDNA. SuperScript III (Invitrogen) was used for cDNA synthesis with 0.5 μM of the reverse primer Env3out (5′-TTGCTACTTGTGATTGCTCCATGT-3′; nucleotides 8913 to 8936 according to HXB2 numbering [88]), as previously described [134]. The cDNA from the reverse transcription reaction served as template for PCR amplification of the 3-kb fragment containing the *env* and *rev* genes. cDNA was serially diluted in water in replicates of three PCR wells and subjected to a nested PCR reaction. The high-fidelity polymerase PrimeStar Max (Takara) was used for first- and second-round PCR reactions. First-round PCR was performed with 1 mM MgCl_2_, 0.2 mM of each dNTP, 0.4 μM of forward primer Env5out (5′-TAGAGCCCTGGAAGCATCCAGGAAG-3′; nucleotides 5853 to 5877), and 0.4 μM of the reverse primer Env3out in an 8-μl reaction mixture. PCR conditions were 94°C for 30 s, followed by 38 cycles of 94°C for 15 s, 55°C for 30 s, and 68°C for 3 min, with a final extension period of 8 min at 68°C. Second-round PCR was performed by diluting the product of the first round 10-fold in water and then transferring 1 μl of the diluted first-round product into a final volume of 8 μl of a reaction mixture containing 0.2 mM of each dNTP, 0.4 μM of forward primer Env5in (5′- GGCATCTCCTATGGCAGGAAGAAG-3′; nucleotides 5960 to 5983), and 0.4 μM of reverse primer Env3in (5′-GTCTCGAGATACTGCTCCCACCC-3′; nucleotides 8904 to 8882). PCR conditions were identical to the first-round PCR. Dilutions that yielded approximately one of three PCR-positive wells were then retested in 12 replicates to identify a dilution in which <30% of wells were positive for amplification products. The amplified fragments from cDNA dilutions in which <30% of wells were positive were then purified from agarose gels using PureLink gel extraction kit (Invitrogen). Purified fragments were cloned into the pSVIIIenv vector [135] using Infusion cloning system (Clontech). All cloned *env* genes were then screened for functionality of their protein product by generating recombinant viruses that contain each Env and testing their infection of CD4^+^CCR5^+^ and CD4^+^CXCR4^+^ cells, as detailed below. Envs that mediated infection of either cell type were further analyzed whereas the remaining Envs were archived for future tests. From each plasma sample, we isolated in the above manner one to eight functional *env* genes.

### Sequence analysis

All functional Envs were fully sequenced (see list of accession numbers in S1 Data). Envs that were identical in amino acid sequence to an already existing Env (16 of 523 functional Envs isolated thus far) were discarded. Similarly, for all clade B and C sequence datasets used to calculate volatility indices, identical protein sequences were excluded. Protein sequences were aligned using a Hidden Markov Model with the HMMER3 software [136]. Since automated algorithms cannot perfectly align Env sequences (mainly because of insertions and deletions), we edited the HMMER3 alignment product manually [137]. For calculation of genetic pairwise distances, gapped sites were not counted in the distance calculations unless present in 95% of the aligned sequences. This cutoff was applied to minimize the false similarities between sequences introduced by gapped sites when using sequences that are highly divergent. The use of multiple sequence alignment for measuring genetic distances between isolates allows rapid calculations. This tool is thus well suited for very large datasets. Nevertheless, genetic distances calculated by this approach are affected by insertions and deletions relative to pairwise alignment tools. We also calculated the volatility indices using a basic pairwise alignment tool (ClustalW). Comparison of the mean volatility indices from 22 patient samples calculated using genetic distances from the multiple sequence and pairwise alignment tools showed limited differences (S7 Fig). The greater precision of pairwise alignments, which allow customization using HIV-specific scoring matrices [138], contributes primarily to analyses that include a small number of patient samples.

Phylogenetic trees were reconstructed from protein sequences using the maximum likelihood method with an HIVb (between patient) amino acid substitution model using PhyML3. Sequence features of Env segments, including length, charge, and number of PNGSs were examined using in-house generated macros for Excel and the “Variable Region Characteristics” tool of the Los Alamos website (hiv.lanl.gov). Boundaries of Env segments (shown in Fig 3D and S8A Fig) conform to the standard segmentation of the *env* gene specified in the above online tool and are based on standard HXBc2 numbering of *env* [88]. PNGSs were defined by presence of the sequence Asn-X-Ser/Thr, in which X can be any amino acid except Pro. The mean hydropathy score for each loop was calculated based on the Black and Mould scale [74], which places values on a scale of 0 to 1.

Sequence analysis of the cloned *envs* suggests that the high-fidelity polymerase used in both rounds of the nested PCR (error rate 7.6 × 10^−7^ by sequencing) introduced minimal errors and therefore had a limited effect on the observed variance in segmental and antigenic features. For the amplification protocol we applied (and given the above error rate), we would expect that ~17% of the 3-kb products would contain an error. Indeed, 16 of the functional Envs we isolated were identical in amino acid sequence to other Envs isolated from the same plasma sample but in different amplification reactions (these Envs were discarded). Twelve additional Envs contain a single amino acid difference from another isolate amplified separately. Therefore, although we do not exclude that some changes may have occurred because of mutations during in vitro amplification, the effect of such changes on sequence and antigenic features is likely minimal.

### Preparation of recombinant luciferase-expressing viruses

Single-round, recombinant HIV-1 viruses that express the luciferase gene were generated by transfection of human embryonic kidney 293T cells (obtained from the American Type Culture Collection, ATCC) using JetPrime transfection reagent (Polyplus). Briefly, cells were seeded in six-well plate wells (8.5 × 10^5^ cells per well) and transfected the next day with 0.4 μg of HIV-1 packaging construct pCMVΔP1ΔenvpA, 1.2 μg of firefly luciferase–expressing construct pHIvec2.luc, 0.4 μg of a plasmid-expressing HIV-1 Env, and 0.2 μg of a plasmid-expressing HIV-1 Rev. On the next day, transfection medium was changed to culture medium (DMEM/10% FBS). Virus-containing supernatants were collected on the following day, cleared of cell debris by low-speed centrifugation, and filtered through 0.45-μm filters. Viruses were then used for infection or snap frozen on dry ice, immersed in ethanol for 15 min, and stored at –80°C until use.

### Infection by luciferase-expressing viruses

Canis familiaris thymus normal (Cf2Th) cells (obtained from the NIH AIDS Reagent Program) expressing CD4 and CCR5 (Cf2Th CD4^+^CCR5^+^) or CD4 and CXCR4 (Cf2Th CD4^+^CXCR4^+^) were used as target cells for measuring infection. Approximately 5 h before infection, cells were detached from culture plates using PBS supplemented with 7.5 mM EDTA and were seeded in 96- or 384-well, luminometer-compatible plates (at a density of 14 or 4.5 × 10^3^ cells per well, respectively). Viruses were then added to the cells and further incubated for 3 d, at which time the medium was removed; cells were lysed with passive lysis buffer (Promega) and subjected to three freeze–thaw cycles. To measure luciferase activity, 100 μl of luciferin buffer (15 mM MgSO_4_, 15 mM KPO_4_ [pH 7.8], 1 mM ATP, and 1 mM dithiothreitol) and 50 μl of 1 mM D-luciferin potassium salt (Syd Labs, MA) were added to each sample in 96-well plates (30 μl and 15 μl, respectively, for samples in 384-well plates). Luminescence was recorded using a Synergy H1 microplate reader (BioTek Instruments).

### Antibodies and other Env probes

The monoclonal antibodies (mAbs) indicated below were obtained through the NIH AIDS Reagent Program, Division of AIDS, NIAID, NIH. The mAb 39F that targets the V3 loop of Env was contributed by James Robinson [72, 139, 140]. The mAb 10E8 that targets the MPER of gp41 was contributed by Mark Connors [68]. Hermann Katinger provided the MPER-targeting mAb 2F5 [70] and mAb 2G12, which targets a carbohydrate-dependent gp120 epitope [65]. The mAb IgG1 b12, which recognizes the CD4-binding site of gp120 [141, 142], was a kind gift from Dennis Burton. The CD4-binding site mAb VRC03 was provided by John Mascola [143]. The International AIDS Vaccine Initiative (IAVI) Neutralizing Antibody Consortium kindly provided mAbs PG9 and PG16 that target overlapping, trimer-dependent epitopes [67] and mAbs PGT121 and PGT126, which target partially overlapping epitopes on gp120 that contain glycan and protein components [63]. The CD4-Ig fusion protein is composed of the Fc region of human IgG1 linked to two copies of the two N-terminal domains of the CD4 molecule. The CD4-Ig protein was produced and purified as previously described [58, 144].

### Cell-based ELISA to measure binding of probes to cell surface–expressed Env

Binding of probes to HIV-1 Env trimers expressed on human osteosarcoma (HOS) cells (obtained from the ATCC) was measured using a modified protocol of the cell-based enzyme-linked immunosorbent assay (ELISA) described previously [58, 59]. Briefly, HOS cells were seeded in 96-well plates (1.2 × 10^4^ cells per well) and transfected after 6 h with 55 ng of a plasmid-expressing Env and 12 ng of a Tat-expressing plasmid per well using 0.18 μl per well of JetPrime (Polyplus Inc.) transfection reagent. For experiments performed in 384-well plates, each well contained 4.5 × 10^3^ cells, which were transfected with 26 ng of an Env-expressing plasmid and 5.5 ng of a Tat-expressing plasmid using 0.08 μl JetPrime reagent. In all experiments, a negative control plasmid was used that contains a stop mutation in amino acid position 46 of Env (according to standard HXBc2 numbering [88]) to determine background binding of each probe to the cells. Three days after transfection, cells were washed twice with blocking buffer (20 mg/ml BSA, 1.8 mM CaCl_2_, 1 mM MgCl_2_, 25 mM Tris [pH 7.5], and 140 mM NaCl) and incubated with the indicated probes in blocking buffer for 45 min. Unless indicated otherwise, all mAbs were added at 0.5 μg/ml whereas CD4-Ig was added at 2 μg/ml. Binding of each probe to the Envs is normalized for the level of Env expression using this saturating concentration of CD4-Ig [58], which binds to the highly conserved CD4-binding site on Env. Relative to other methods of normalization for the level of expression (e.g., by polyclonal sera from multiple patients), the use of CD4-Ig is less affected by the variable antigenicity of the different isolates [111]. All samples were then washed six times with blocking buffer and incubated with a horseradish peroxidase (HRP)-conjugated goat antihuman IgG polyclonal antibody preparation for 45 min. Cells were subsequently washed six times with blocking buffer and six times with washing buffer (140 mM NaCl, 1.8 mM CaCl_2_, 1 mM MgCl_2_, and 20 mM Tris [pH 7.5]). HRP enzyme activity was determined after addition of 35 μl per well of a 1:1 mix of SuperSignal West Pico Chemiluminescent peroxide and luminol enhancer solutions (Thermo Fisher Scientific) supplemented with 150 mM NaCl. To samples in 384-well plates we added 25 μl of the reagent mix. Light emission was measured with a Synergy H1 reader.

### Data processing, archiving, and statistical analyses

All software for the Data Processing, Archiving and Exploration Platform was custom developed in-house with the software company Bio::Neos (Coralville, IA). Cell-based ELISA measurements (exported from the luminometer and expressed as relative light units [RLUs]) are processed by the software. Reliability indices are assigned to each set based on the expression level of the Env, the quality of the reads for the negative and positive controls, and the measured variance between the three replicates tested. Binding values are then associated with each Env and stored in a MySQL database. All features of each Env and experimental results can be queried and exported using a graphical user interface (GUI). The amino acid sequence of each Env is also archived in the database, allowing querying of different sequence and segmental features. Analyses of antigenic and segmental data were performed using in-house-designed Excel VBA macros. The K^2^ Omnibus statistic of the D'Agostino and Pearson test was calculated using GraphPad Prism version 6.00 for Windows (Graphpad Software). All other statistics, including Levene’s test and Generalized Estimation Equations (GEE), were performed using R Studio Version 2.11.1 with the car and geepack software packages, respectively. GEE was performed by defining the feature value as the dependent variable, which is approximated by the Period (i.e., Period1 or Period3). Calculations were performed by using an identity vector to cluster unique patients and a Gaussian function for link and variance, with an exchangeable correlation structure.

### Correction of antigenicity data by a logistic function

The numerical output of the cell-based ELISA spans a range of five orders of magnitude. Data are normalized for the cell-surface expression levels of each Env using the CD4-Ig probe and are expressed as percent binding of the probe to the control AD8 Env [111]. Our previous work has shown that the biological relevance of a given fold-change in binding efficiency is not identical throughout the 5-log dynamic range (e.g., the interval between 10% and 100% is not equivalent to the interval between 0.01 and 0.1%) [59, 145]. Based on these studies, we determined the parameters for a logistic function that “trims” lower and upper extreme values:

$$x_{c}=\;\left(\frac{4}{1-e^{-k\left(x-2\right)}}\right)$$ (8)

where *x*_*c*_ is the logistic function–corrected value, *k* is the slope (calculated as 0.6), and *x* is the log-transformed binding value. The comparison between distribution of the raw (log-transformed) and logistic function–corrected data is shown in S2 Fig.

## Supporting information

S1 Fig Phylogenetic tree of Envs included in this study. The tree was reconstructed from protein sequences using the maximum likelihood method and is rooted to the clade B consensus sequence (labeled in black). Envs labeled in red were isolated from samples collected in Iowa City. Envs labeled in blue were isolated from samples collected in Seattle. The HXB2 Env is labeled in black. All Envs belong to viruses from clade B except Envs from patients IC.798 and IC.999, which belong to clades A and AD, respectively. Amino acid sequence alignment of the Envs is provided in S3 Data.
(PDF)

S2 Fig Distribution of antigenicity feature values in Envs isolated from samples collected in Iowa City and Seattle. To avoid sampling bias, each of the 120 patients is represented by a maximum of two Envs per sample. For each longitudinal patient we selected only one plasma sample. The top histogram describes the log-transformed binding values, expressed as percent binding of the probe to the AD8 Env and normalized for cell-surface expression using CD4-Ig. The bottom histogram describes the data after applying the logistic function, which is aimed at reducing the effects of very low and high values, to define the biologically-relevant dynamic range (see Materials and methods section). Data underlying this figure can be found in S6 Data.
(PDF)

S3 Fig Historic changes in antigenic features of Envs isolated from samples collected in Iowa City and Seattle. The *p*-values describing equality of the means in Iowa City samples from Period1 (27 patients) and Period3 (30 patients) were calculated using generalized estimating equations (GEE), which accounts for differential sampling (plasma samples and Envs) from each patient [146] (labeled P_*μ*_). Equality of variances between Period1 (1985–1991) and Period3 (2005–2012) was calculated using Levene’s test. The *p*-value for the null hypothesis of equal variance is labeled P_var_ and is highlighted in a color that describes its statistical significance (green, high; red, low). To calculate P_var_, each patient was represented by a single value (an average was first calculated for all Envs in each sample and the values obtained for all samples of the patient within that period were averaged). Detailed description of these calculations is provided in the **Materials and Methods** section. To examine historic changes in epitope integrity we sectioned the time period into 5–6 year groups. For each sub-period we quantified the percentage of Envs that bind the probe inefficiently (marked by green circles), which is defined as less than 5% of probe binding to the control AD8 Env. Data underlying this figure can be found in S6 Data.
(PDF)

S4 Fig Historic changes in segmental features of the five variable loops of Env measured in samples collected in Iowa City and Seattle. Data represent amino acid length, mean hydropathy score (measured by the Black and Mould scale), net charge and total number of Potential N-linked glycosylation sites (PNGS) of the five variable loops. The *p*-values for equality of the means and variance tests between Period1 and Period3 were calculated using GEE and Levene’s test, as outlined in S3 Fig and are labeled P_*μ*_ and P_var_, respectively. Changes in Iowa City were calculated using data from 32 and 31 patients from Period1 and Period3, respectively. Data underlying this figure can be found in S6 Data.
(PDF)

S5 Fig Population-level changes in diversity of Env segmental and antigenic features. **(A)** Comparison between diversity of length, hydropathy, charge and PNGS of the 23 listed segments of Env. The Period1 and Period3 panels are composed of 32 and 31 patients, respectively. Diversity of segmental feature values was higher during Period3 than Period1 (*p*-value of 0.0032 was calculated in a paired T-test). **(B)** Comparison between diversity of antigenic features of Env in samples collected during Period1 (27 patients) and Periods3 (30 patients). SP, signal peptide; C, constant region; V, variable loop; FP, fusion peptide; HR1 and HR2, heptad repeat regions 1 and 2; MPER, membrane proximal ectodomain region; TM, transmembrane domain; Endo, endodomain of gp41; KEN, Kennedy epitope region; LLP, lentiviral lytic peptide regions 1–3. Data underlying this figure can be found in S6 Data.
(PDF)

S6 Fig In-host variance of segmental features of the five variable loops measured using the cross-sectional panel of samples (from 60 patients). Values represent the variance measured in each feature value among the different Envs isolated from the same plasma sample, as calculated by the coefficient of variation (CoV). The CoV values are color-coded according to their values (white, low; purple, high). Data underlying this figure can be found in S6 Data.
(PDF)

S7 Fig Effect of method to measure genetic distances on the calculated volatility index. **(A)** Protein sequences of all Envs from Iowa City or Seattle were aligned using the multiple sequence alignment tool HMMER3, as described in the **Materials and Methods** section, and genetic distances between all Envs contained in each sample were calculated. In addition, we performed pairwise alignments for all Envs contained in each of 22 plasma samples using the ClustalW tool. Data represent the correlation between the genetic distances calculated using the two methods. **(B)** The Volatility Indices of length, hydropathy score, charge and PNGS were calculated for the 23 segments of Env using genetic distances obtained from the multiple sequence alignment and the pairwise alignment methods. The mean Volatility Index of the 22 plasma samples as calculated by the two methods is compared. Data underlying this figure can be found in S6 Data.
(PDF)

S8 Fig Volatility indices of segmental features of Env. **(A)** Mean Volatilities measured using samples collected in Iowa City from 60 cross-sectional patients. SP, signal peptide; C, constant region; V, variable loop; FP, fusion peptide; HR1 and HR2, heptad repeat regions 1 and 2; MPER, membrane proximal ectodomain region; TM, transmembrane domain; Endo, endodomain of gp41; KEN, Kennedy epitope region; LLP, lentiviral lytic peptide regions 1–3. **(B)** Correlation between Volatility Indices of hydropathy, charge, length and PNGS of 23 segments of Env using plasma samples collected in Iowa City during Period1 and Period3. **(C)** Correlation between Volatility Indices of antigenicity features calculated using samples collected during Period1 and Period3. Data underlying this figure can be found in S6 Data.
(PDF)

S9 Fig Longitudinal divergence of variable loop features. **(A)** Data represent the phenotypic and genetic pairwise distances between the reference isolates and all other Envs from each patient, as described in the legend to Fig 4. To monitor the progression of variance and allow equal representation for all patients we divided the x-axis into sections of 0.01 genetic distance units (see vertical lines). For each section, all phenotypic pairwise distances from the same patient were averaged. The variance among different patient averages for the same section was then calculated (labeled by red squares). Due to the small number of isolates in sections that describe larger genetic distances, calculations were performed only for sections of 0.01 to 0.09 distance units. **(B)** Comparison between the Volatility Index of the indicated features of the five variable loops and their mean longitudinal divergence in 18 patients calculated using a linear regression model. For each patient we measured the phenotypic distances that separate all Envs from the reference isolate/s and data were plotted against the genetic distance. The mean divergence (change in feature value per genetic distance unit) was computed for each patient by fitting a linear regression model to all data points. Values from all longitudinal patients were then averaged. Data underlying this figure can be found in S6 Data.
(PDF)

S10 Fig Longitudinal divergence of antigenic features of Env in 18 patients monitored for up to 11 years. Data represent the phenotypic pairwise distances between each reference isolate and all other Envs from that patient and are divided by the value of the reference isolate. Red squares describe the variance in feature values among patients, as calculated for each genetic distance section (see legend to S9 Fig). Data underlying this figure can be found in S6 Data.
(PDF)

S11 Fig Distribution of segmental features of the gp120 variable loops in Envs isolated from samples collected in Iowa City and Seattle. Insets indicate values of the K^2^ Omnibus statistic of the D'Agostino and Pearson test, which describes departure from normality of the distribution. The K^2^ values are color labeled according to their values (green, significant departure; red, normally distributed). Statistical significance of the departure is directly related to the K^2^ Omnibus statistic (in this test all values >6 were associated with a *p*-value <0.05). For example, length of the V1 loop is normally distributed in the population, whereas length of the V2 loop shows significant departure from normality (see sharp decline in frequency of Envs with V2 loops shorter than 38 amino acids). Primary data are provided in S1 and S6 Data.
(PDF)

S12 Fig Relationship between volatility and population diversity of the indicated segmental features of Env. Volatility was calculated using Envs from the 20 plasma samples of the MOTIVATE trial. Diversity was calculated by the standard deviation of the values among plasma samples collected in Iowa City during Period3 (2005–2012). SP, signal peptide; V, variable loop; C, constant region. Data underlying this figure can be found in S6 Data.
(PDF)

S13 Fig Schematic of the volatility-based approach to calculate the predicted range of amino acid variants that emerge in the population. An example is given of calculating the likelihood of Alanine appearing at a given position when the reference sequence contained Proline. Volatility of charge, molecular weight and hydropathy was calculated for the position. For convenience, molecular weight was converted to a scale of 0 to 1. For each feature type, a probability density function was generated that describes the likelihood of change to each amino acid based on the measured Volatility of the feature at this position, the ancestral state (i.e., Proline) and query (i.e., Alanine in this example). The combined likelihood based on Volatilities of the three feature types was calculated as the product of the three functions.
(PDF)

S14 Fig Distribution of the pairwise genetic distances between Envs isolated from the same plasma sample. Data describe Envs isolated from all longitudinal and cross-sectional plasma samples collected in Iowa City and Seattle. Data underlying this figure can be found in S6 Data.
(PDF)

S1 Data Antigenic and segmental features of Envs isolated from cross-sectional and longitudinal samples collected in Iowa City and Seattle. **(A)** Binding of the indicated probes to each Env was measured using a cell-based ELISA system. Data are expressed as percent binding of the antibody to the tested Env relative to its binding to the control AD8 Env. Binding is normalized for the level of cell-surface expression using saturating concentrations of CD4-Ig [58]. We note that for analysis of these data, throughout this work all values were log-transformed and corrected by a logistic function (see Materials and methods section and S2 Fig). **(B)** Segmental features of Envs analyzed. For each segment we calculated the amino acid length (L), number of PNGS (G), Charge (C) and mean hydropathy score (H). Amino acid positions of each segment (numbered according to the HXBc2 convention [88]) are indicated in S5A Fig. The accession number of each Env and year of isolation are indicated. N/A, Genbank accession number not yet assigned. ND, test not performed.
(XLSX)

S2 Data Raw data underlying plots and graphs in Figs 1–7. (XLSX)

S3 Data Amino acid sequence alignment (FASTA format) of 523 Envs we isolated from plasma samples collected in Iowa City and Seattle. All Envs are aligned against the HXBc2 isolate.
(FAS)

S4 Data Amino acid sequence alignment (FASTA format) of Envs from the MOTIVATE trial used for measuring the volatility index of segmental and sequence features. For clade B and C datasets used to calculate Volatility indices, identical protein sequences were excluded. A single isolate (accession number KT452440) was excluded from the MOTIVATE trial dataset due to multiple mutations in gp120 and gp41 that are atypical of all HIV-1 groups and unique to this isolate relative to all Envs from this patient. All Envs were isolated from plasma samples collected before initiation of Maraviroc treatment.
(FAS)

S5 Data Amino acid sequence alignment (FASTA format) of Envs of clade C viruses used for analysis of the volatility index of segmental features (Panel#1 and Panel#2 in Fig 4D). All Envs included in the alignment were isolated from plasma samples.
(FAS)

S6 Data Raw data underlying plots and graphs in supporting information figures. Raw Data for supporting information S2, S3, S4, S5, S6, S7, S8, S9, S10, S11, S12 and S14 Figs.
(XLSX)

## Abbreviations

ART: antiretroviral therapy
C: constant region
CoV: coefficient of variation
Env: envelope glycoprotein
mAb: monoclonal antibody
MPER: membrane-proximal ectodomain region
NS: not statistically significant
PNGS: potential N-linked glycosylation site
SGA: single genome amplification
SP: signal peptide
V: variable loop
