# Supplementary figures and images for: Accurate predictions of population-level changes in sequence and structural properties of HIV-1 Env using a volatility-controlled diffusion model
Source: PLoS Biol. 2017 Apr 6;15(4):e2001549. doi: 10.1371/journal.pbio.2001549 (PMC5383018; doi:10.1371/journal.pbio.2001549)

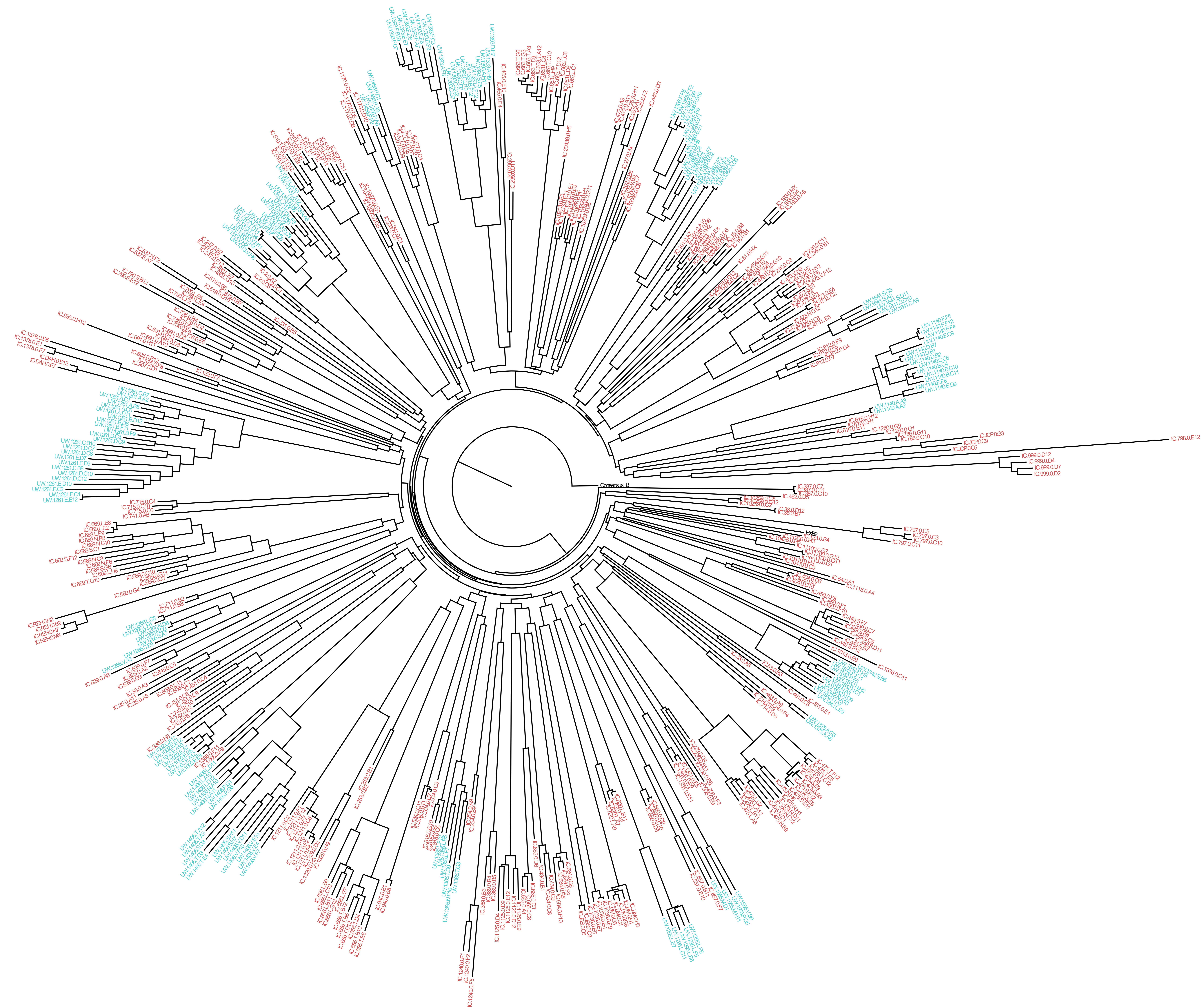

0.04

Supplement: S1 Fig — The tree was reconstructed from protein sequences using the maximum likelihood method and is rooted to the clade B consensus sequence (labeled in black). Envs labeled in red were isolated from samples collected in Iowa City. Envs labeled in blue were isolated from samples collected in Seattle. The HXB2 Env is labeled in black. All Envs belong to viruses from clade B except Envs from patients IC.798 and IC.999, which belong to clades A and AD, respectively. Amino acid sequence alignment of the Envs is provided in S3 Data. (PDF) [file pbio.2001549.s001.pdf]

Frequency

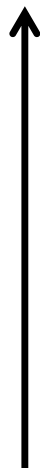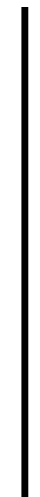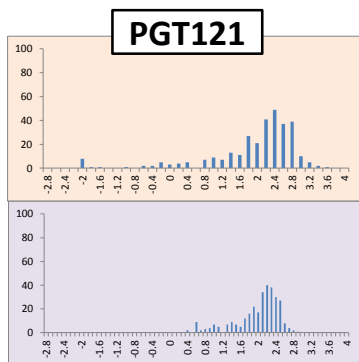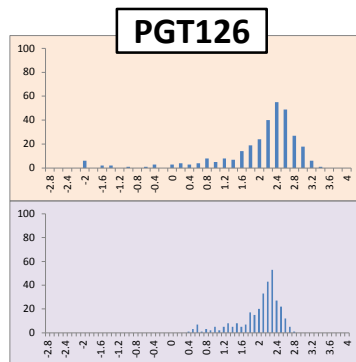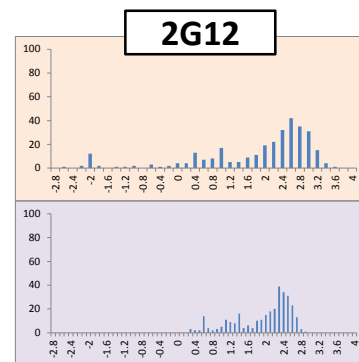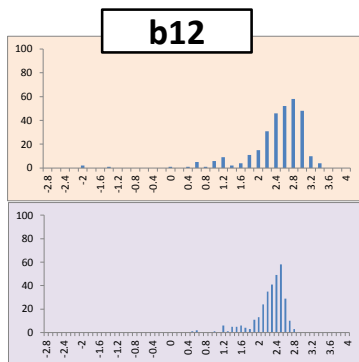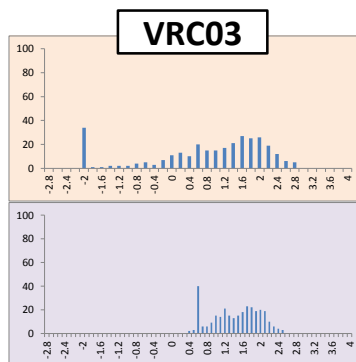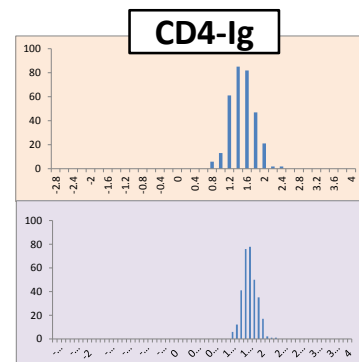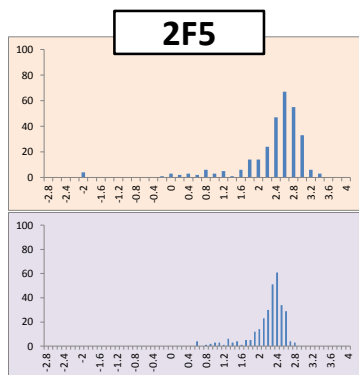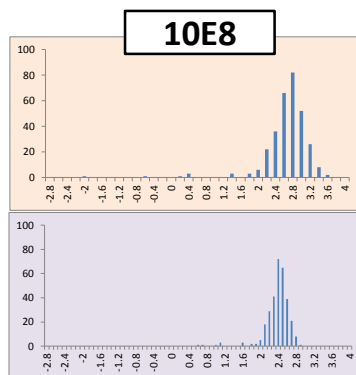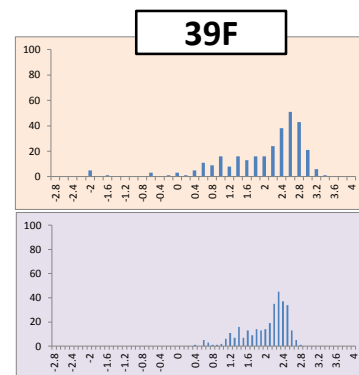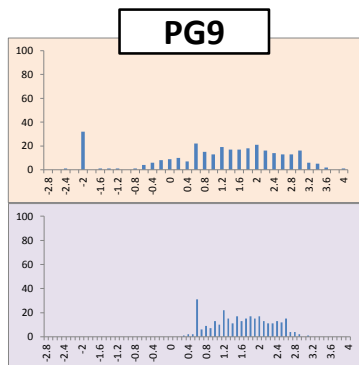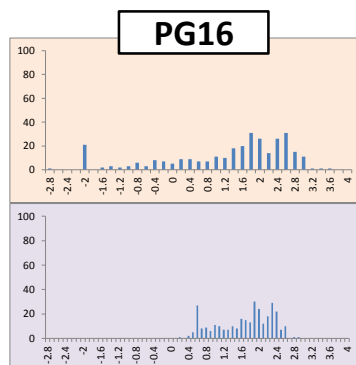

Log-transformed  
values

Logistic function-  
corrected values

Probe Binding

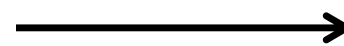

Supplement: S2 Fig — To avoid sampling bias, each of the 120 patients is represented by a maximum of two Envs per sample. For each longitudinal patient we selected only one plasma sample. The top histogram describes the log-transformed binding values, expressed as percent binding of the probe to the AD8 Env and normalized for cell-surface expression using CD4-Ig. The bottom histogram describes the data after applying the logistic function, which is aimed at reducing the effects of very low and high values, to define the biologically-relevant dynamic range (see Materials and methods section). Data underlying this figure can be found in S6 Data. (PDF) [file pbio.2001549.s002.pdf]

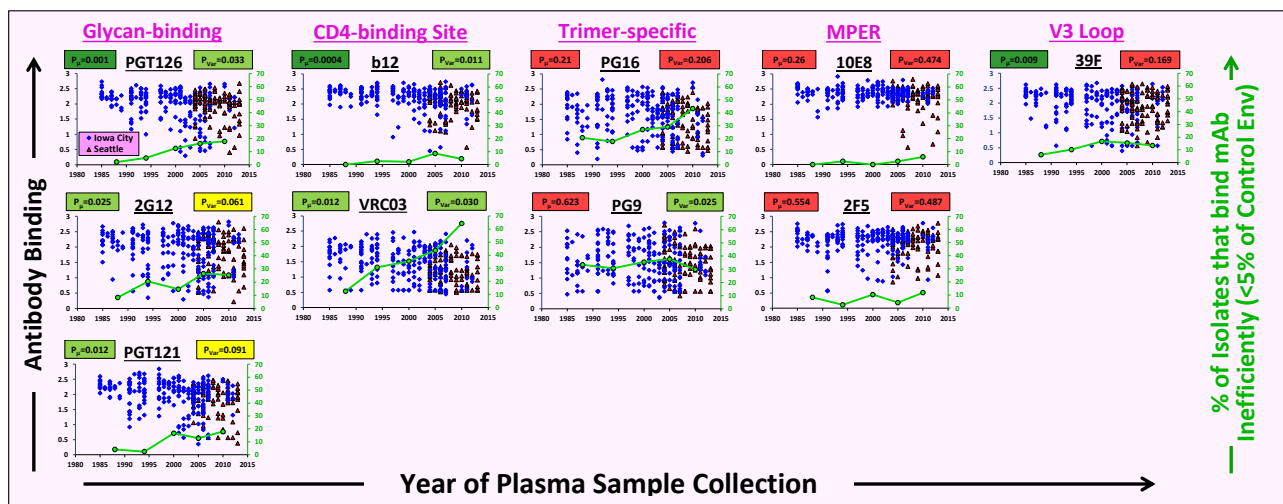

Supplement: S3 Fig — The p-values describing equality of the means in Iowa City samples from Period1 (27 patients) and Period3 (30 patients) were calculated using generalized estimating equations (GEE), which accounts for differential sampling (plasma samples and Envs) from each patient [146] (labeled Pμ). Equality of variances between Period1 (1985–1991) and Period3 (2005–2012) was calculated using Levene’s test. The p-value for the null hypothesis of equal variance is labeled Pvar and is highlighted in a color that describes its statistical significance (green, high; red, low). To calculate Pvar, each patient was represented by a single value (an average was first calculated for all Envs in each sample and the values obtained for all samples of the patient within that period were averaged). Detailed description of these calculations is provided in the Materials and Methods section. To examine historic changes in epitope integrity we sectioned the time period into 5–6 year groups. For each sub-period we quantified the percentage of Envs that bind the probe inefficiently (marked by green circles), which is defined as less than 5% of probe binding to the control AD8 Env. Data underlying this figure can be found in S6 Data. (PDF) [file pbio.2001549.s003.pdf]

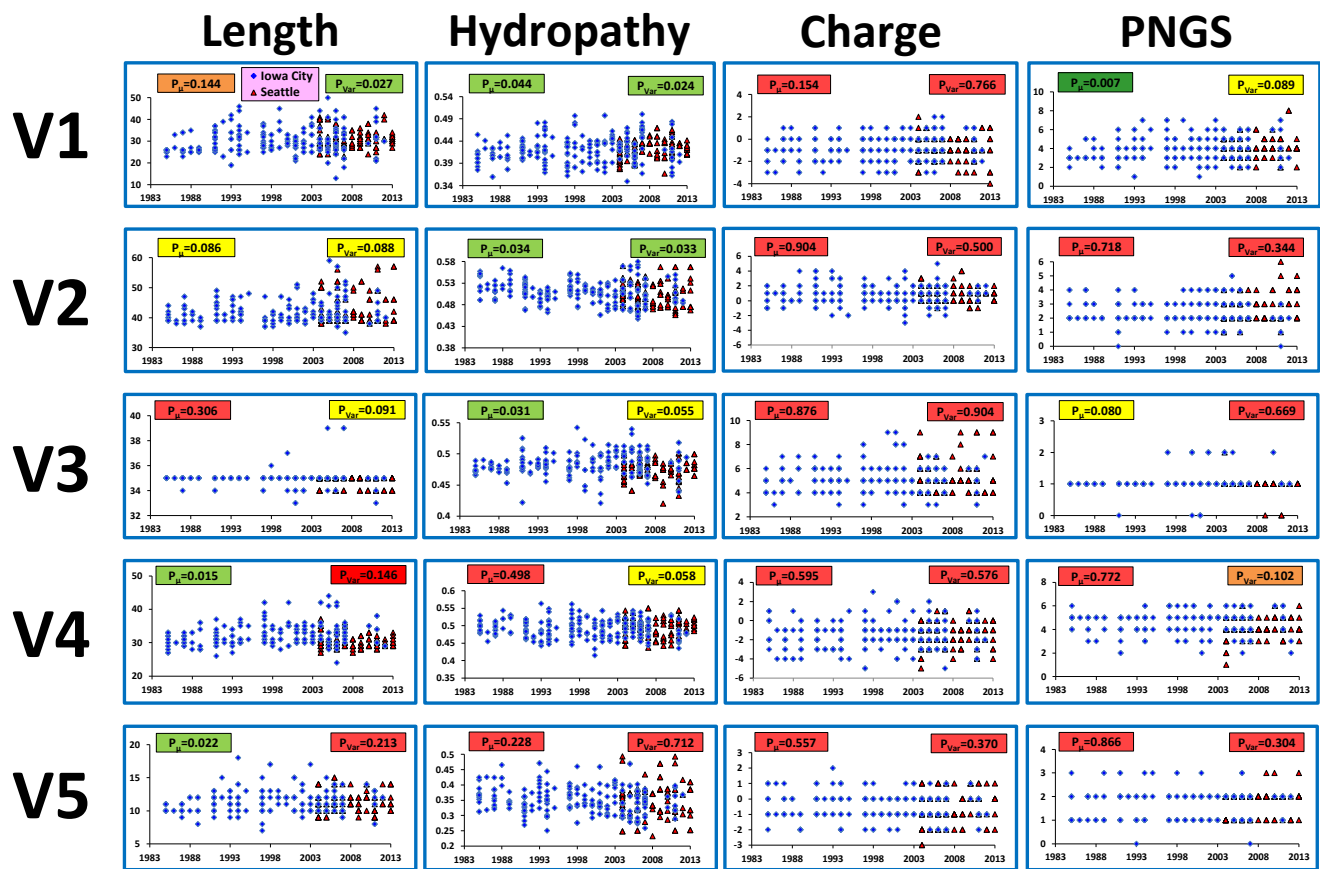

Supplement: S4 Fig — Data represent amino acid length, mean hydropathy score (measured by the Black and Mould scale), net charge and total number of Potential N-linked glycosylation sites (PNGS) of the five variable loops. The p-values for equality of the means and variance tests between Period1 and Period3 were calculated using GEE and Levene’s test, as outlined in S3 Fig and are labeled Pμ and Pvar, respectively. Changes in Iowa City were calculated using data from 32 and 31 patients from Period1 and Period3, respectively. Data underlying this figure can be found in S6 Data. (PDF) [file pbio.2001549.s004.pdf]

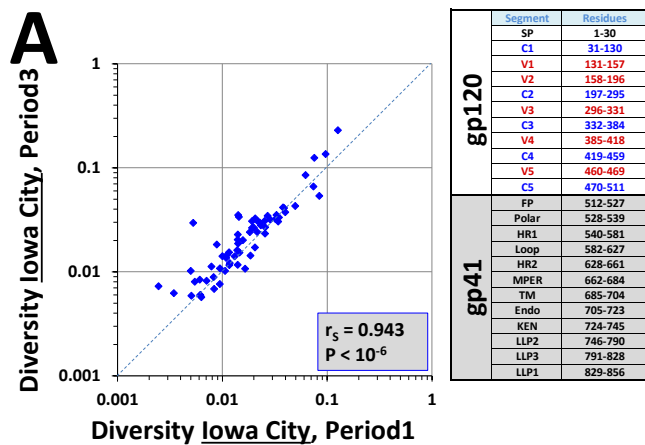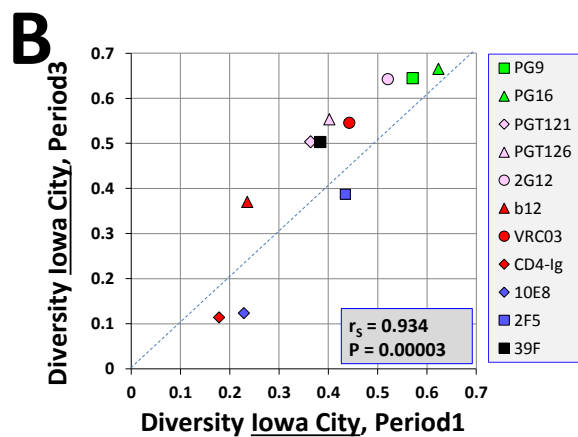

Supplement: S5 Fig — (A) Comparison between diversity of length, hydropathy, charge and PNGS of the 23 listed segments of Env. The Period1 and Period3 panels are composed of 32 and 31 patients, respectively. Diversity of segmental feature values was higher during Period3 than Period1 (p-value of 0.0032 was calculated in a paired T-test). (B) Comparison between diversity of antigenic features of Env in samples collected during Period1 (27 patients) and Periods3 (30 patients). SP, signal peptide; C, constant region; V, variable loop; FP, fusion peptide; HR1 and HR2, heptad repeat regions 1 and 2; MPER, membrane proximal ectodomain region; TM, transmembrane domain; Endo, endodomain of gp41; KEN, Kennedy epitope region; LLP, lentiviral lytic peptide regions 1–3. Data underlying this figure can be found in S6 Data. (PDF) [file pbio.2001549.s005.pdf]

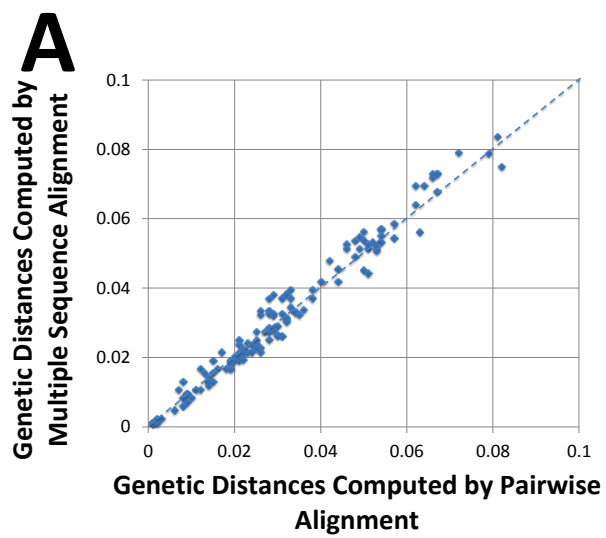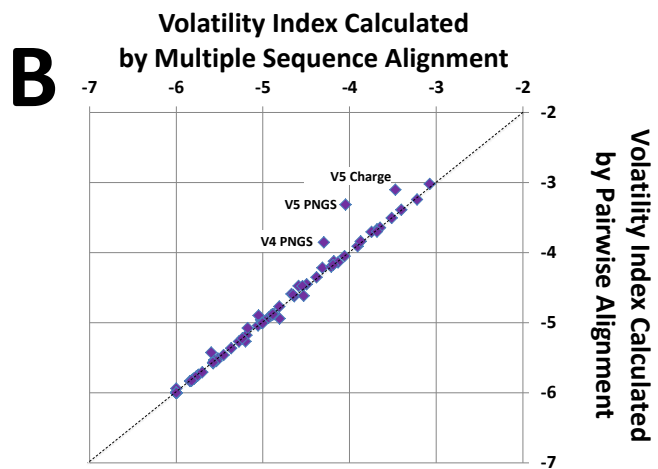

Supplement: S7 Fig — (A) Protein sequences of all Envs from Iowa City or Seattle were aligned using the multiple sequence alignment tool HMMER3, as described in the Materials and Methods section, and genetic distances between all Envs contained in each sample were calculated. In addition, we performed pairwise alignments for all Envs contained in each of 22 plasma samples using the ClustalW tool. Data represent the correlation between the genetic distances calculated using the two methods. (B) The Volatility Indices of length, hydropathy score, charge and PNGS were calculated for the 23 segments of Env using genetic distances obtained from the multiple sequence alignment and the pairwise alignment methods. The mean Volatility Index of the 22 plasma samples as calculated by the two methods is compared. Data underlying this figure can be found in S6 Data. (PDF) [file pbio.2001549.s007.pdf]

# A

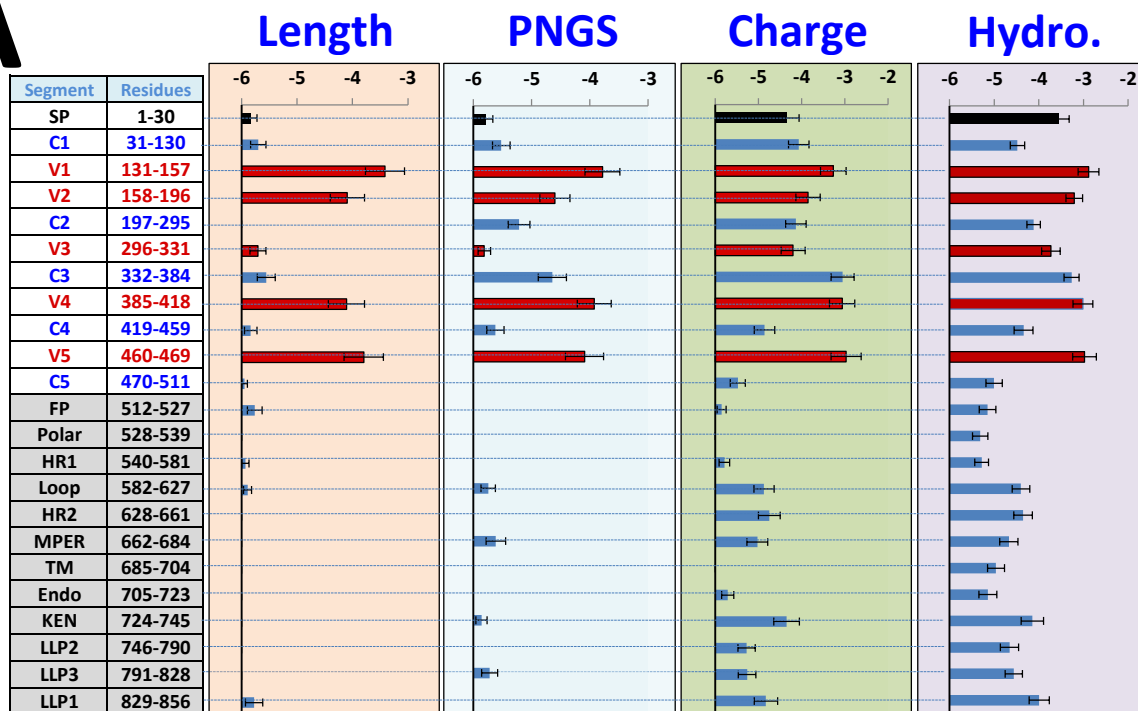

# B

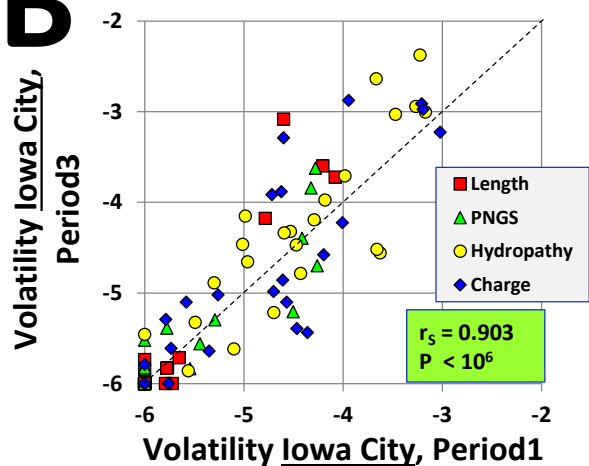

# C

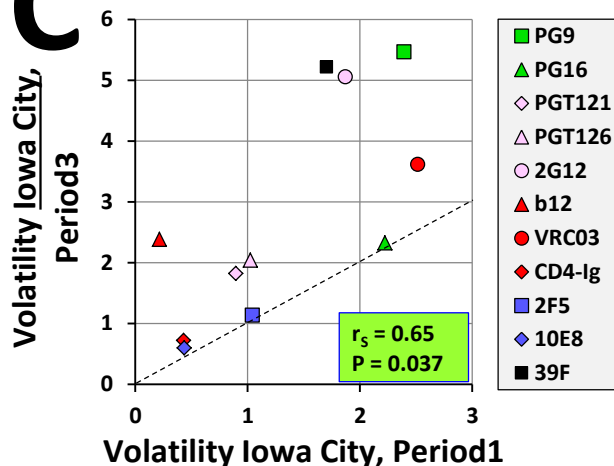

Supplement: S8 Fig — (A) Mean Volatilities measured using samples collected in Iowa City from 60 cross-sectional patients. SP, signal peptide; C, constant region; V, variable loop; FP, fusion peptide; HR1 and HR2, heptad repeat regions 1 and 2; MPER, membrane proximal ectodomain region; TM, transmembrane domain; Endo, endodomain of gp41; KEN, Kennedy epitope region; LLP, lentiviral lytic peptide regions 1–3. (B) Correlation between Volatility Indices of hydropathy, charge, length and PNGS of 23 segments of Env using plasma samples collected in Iowa City during Period1 and Period3. (C) Correlation between Volatility Indices of antigenicity features calculated using samples collected during Period1 and Period3. Data underlying this figure can be found in S6 Data. (PDF) [file pbio.2001549.s008.pdf]

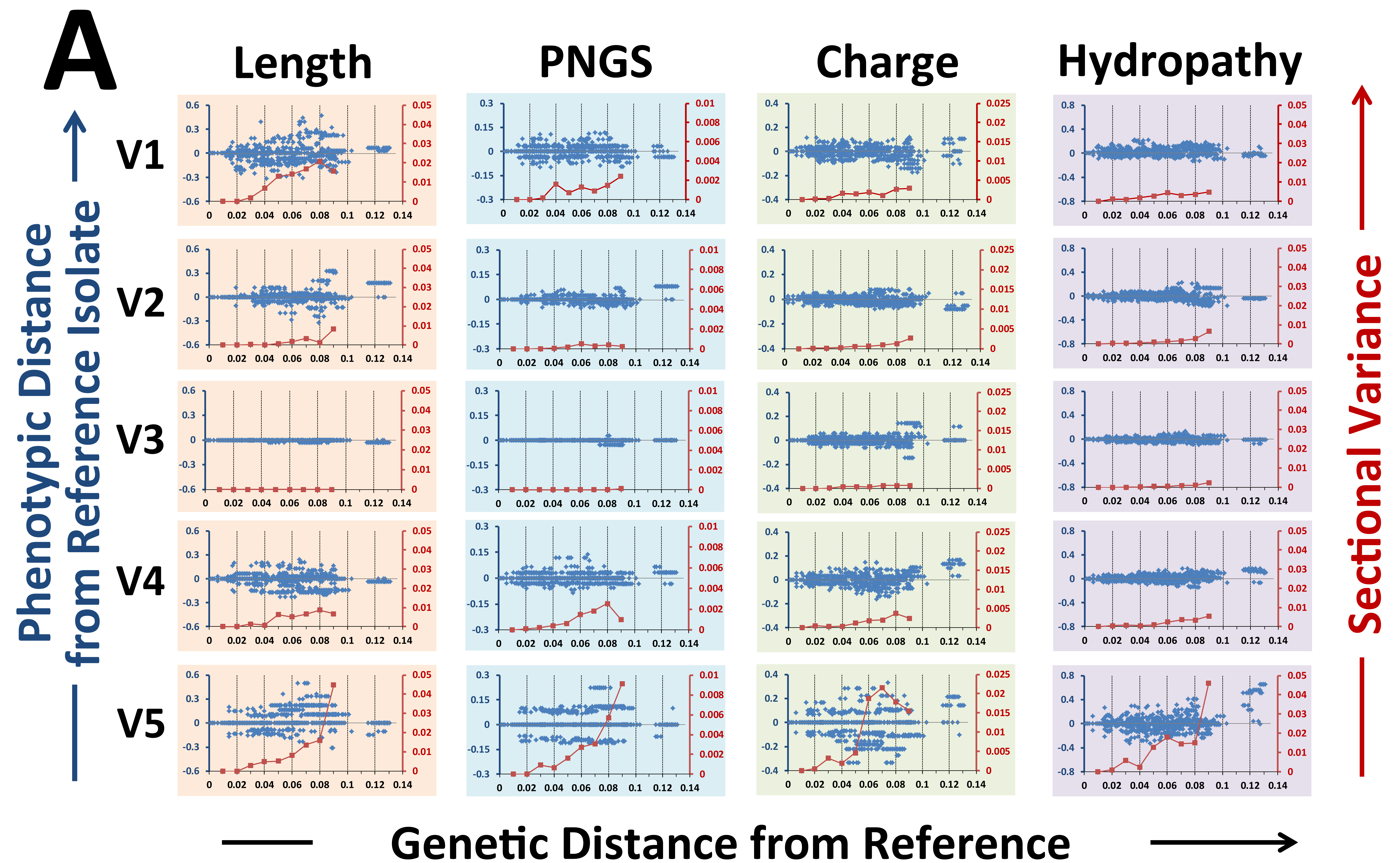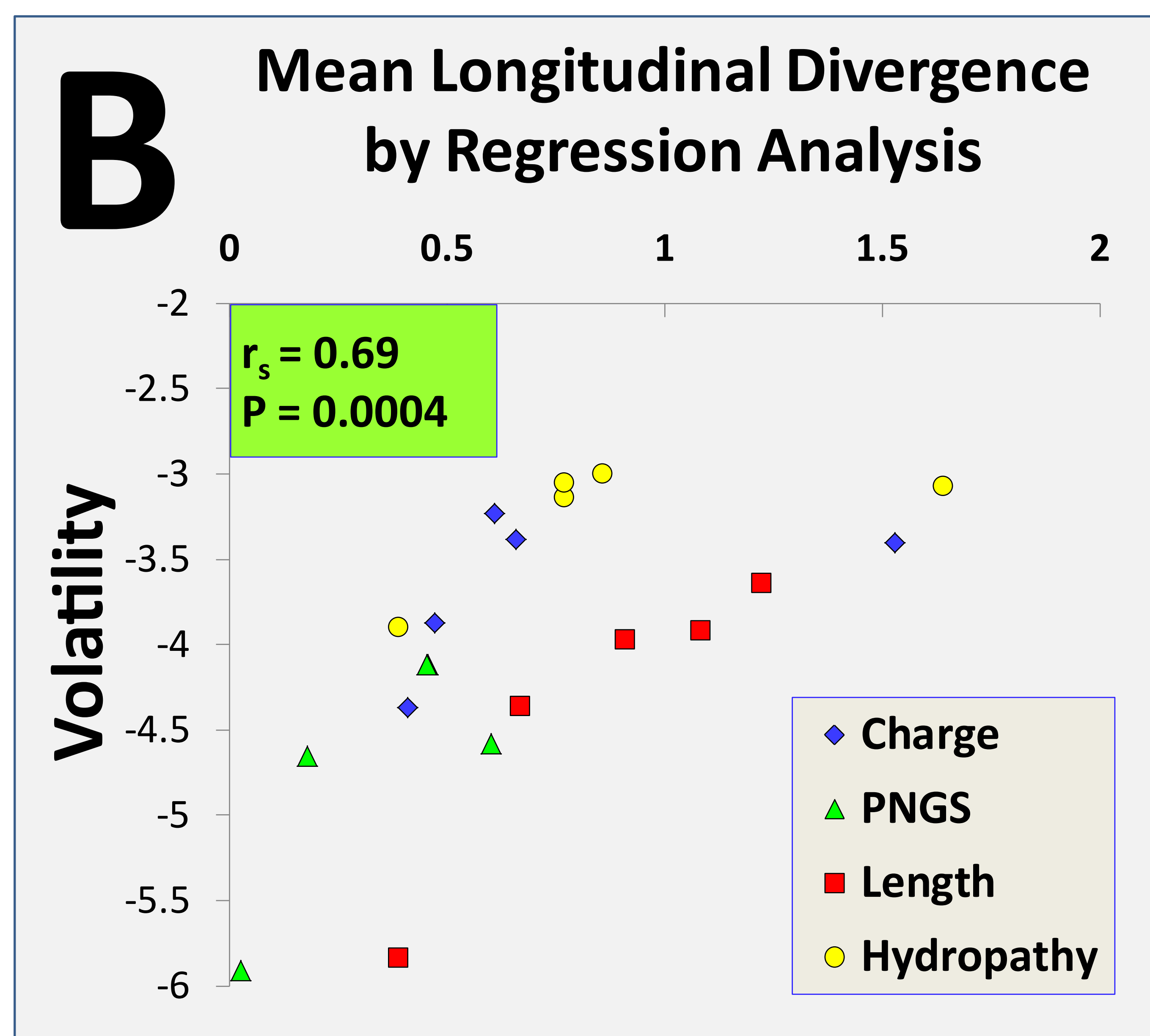

Supplement: S9 Fig — (A) Data represent the phenotypic and genetic pairwise distances between the reference isolates and all other Envs from each patient, as described in the legend to Fig 4. To monitor the progression of variance and allow equal representation for all patients we divided the x-axis into sections of 0.01 genetic distance units (see vertical lines). For each section, all phenotypic pairwise distances from the same patient were averaged. The variance among different patient averages for the same section was then calculated (labeled by red squares). Due to the small number of isolates in sections that describe larger genetic distances, calculations were performed only for sections of 0.01 to 0.09 distance units. (B) Comparison between the Volatility Index of the indicated features of the five variable loops and their mean longitudinal divergence in 18 patients calculated using a linear regression model. For each patient we measured the phenotypic distances that separate all Envs from the reference isolate/s and data were plotted against the genetic distance. The mean divergence (change in feature value per genetic distance unit) was computed for each patient by fitting a linear regression model to all data points. Values from all longitudinal patients were then averaged. Data underlying this figure can be found in S6 Data. (PDF) [file pbio.2001549.s009.pdf]

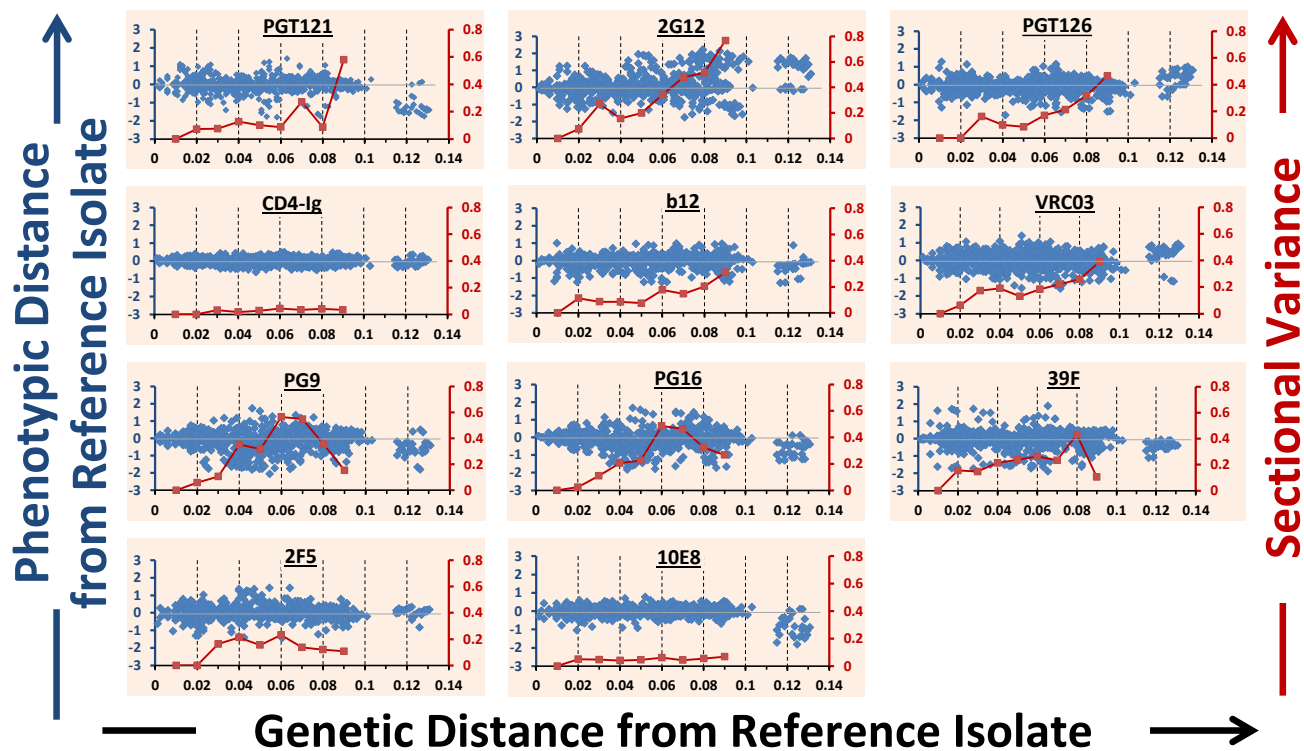

Supplement: S10 Fig — Data represent the phenotypic pairwise distances between each reference isolate and all other Envs from that patient and are divided by the value of the reference isolate. Red squares describe the variance in feature values among patients, as calculated for each genetic distance section (see legend to S9 Fig). Data underlying this figure can be found in S6 Data. (PDF) [file pbio.2001549.s010.pdf]

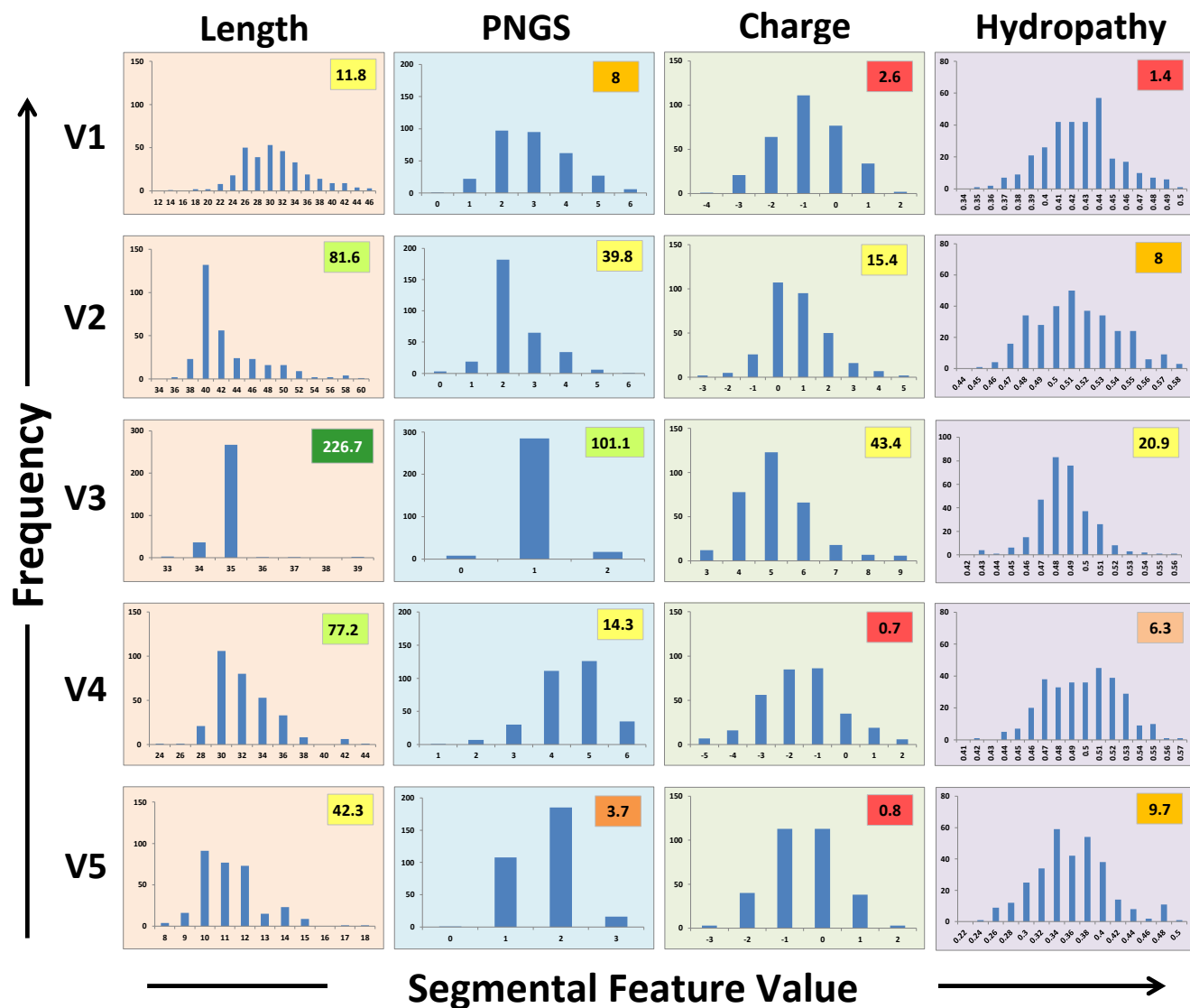

Supplement: S11 Fig — Insets indicate values of the K2 Omnibus statistic of the D'Agostino and Pearson test, which describes departure from normality of the distribution. The K2 values are color labeled according to their values (green, significant departure; red, normally distributed). Statistical significance of the departure is directly related to the K2 Omnibus statistic (in this test all values >6 were associated with a p-value <0.05). For example, length of the V1 loop is normally distributed in the population, whereas length of the V2 loop shows significant departure from normality (see sharp decline in frequency of Envs with V2 loops shorter than 38 amino acids). Primary data are provided in S1 and S6 Data. (PDF) [file pbio.2001549.s011.pdf]

Feature Diversity, Period3

Length

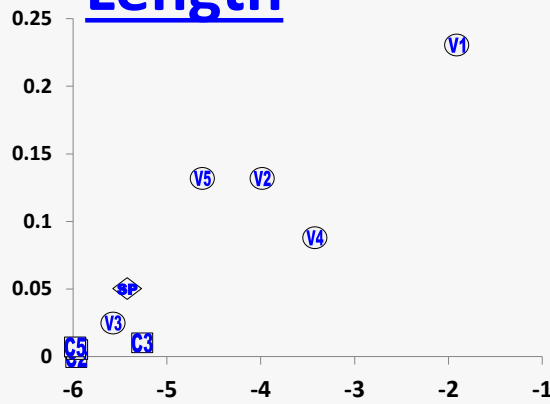

Hydropathy

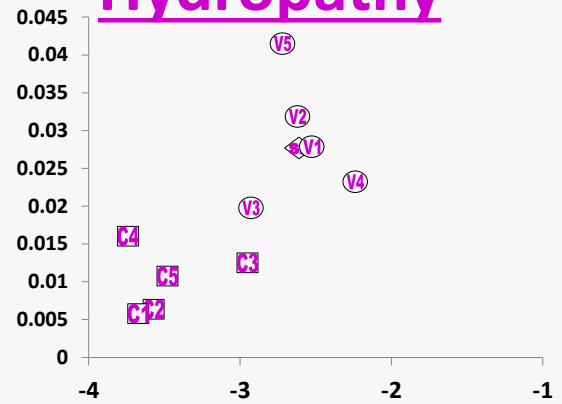

PNGS

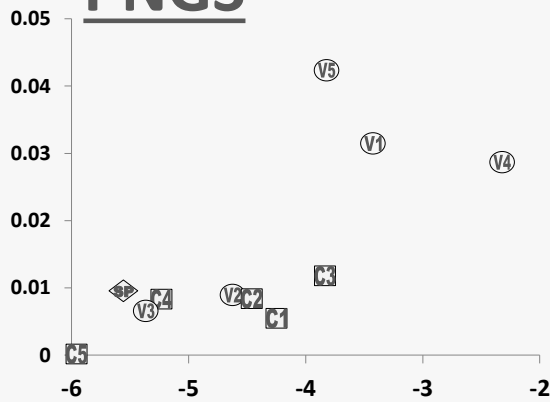

Charge

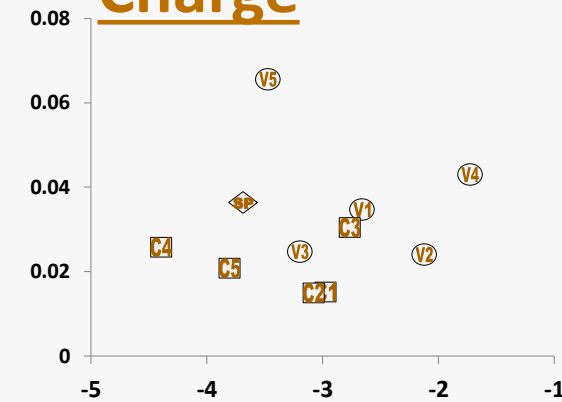

Feature Volatility

Supplement: S12 Fig — Volatility was calculated using Envs from the 20 plasma samples of the MOTIVATE trial. Diversity was calculated by the standard deviation of the values among plasma samples collected in Iowa City during Period3 (2005–2012). SP, signal peptide; V, variable loop; C, constant region. Data underlying this figure can be found in S6 Data. (PDF) [file pbio.2001549.s012.pdf]
