# Supplementary material for: Accurate predictions of population-level changes in sequence and structural properties of HIV-1 Env using a volatility-controlled diffusion model
Source: PLoS Biol. 2017 Apr 6;15(4):e2001549. doi: 10.1371/journal.pbio.2001549 (PMC5383018; doi:10.1371/journal.pbio.2001549)
Supplement: S6 Fig — Values represent the variance measured in each feature value among the different Envs isolated from the same plasma sample, as calculated by the coefficient of variation (CoV). The CoV values are color-coded according to their values (white, low; purple, high). Data underlying this figure can be found in S6 Data. (PDF) [file pbio.2001549.s006.pdf]

	Length					PNGS					Charge					Hydropathy				
**Patient**	**V1**	**V2**	**V3**	**V4**	**V5**	**V1**	**V2**	**V3**	**V4**	**V5**	**V1**	**V2**	**V3**	**V4**	**V5**	**V1**	**V2**	**V3**	**V4**	**V5**
IC.10048	0	0	0	0	0	0.283	0	0	0	0.283	0.471	1.414	0	0	0.471	0.061	0.015	0.009	0.006	0.037
IC.10196	0.047	0	0	0	0.056	0.185	2E-16	0	0	0.054	3.258	2E-16	0.108	0	0.054	0.02	0	0.007	0	0.011
IC.102	0	0	0	0	0	0.283	0	0	0	0	0	0	0	0	0	0.009	0.009	0	0	0
IC.10213	0	0	0	0.02	0	1E-16	2E-16	0	0.134	0.433	0	2E-16	0	0.237	0.866	2E-16	0	1E-16	0.051	0.028
IC.10259	0	0.014	0	0	0.112	0	0.205	0	0	0.119	0.217	0.014	0	0	0.866	0.029	0.009	0	0	0.012
IC.1036	0.23	0	0	0	0.122	0.147	0	0	0	0.192	0.667	2	0.065	0	0.14	0.023	0.014	0.016	0	0.031
IC.10419	0	0	0	0	0	0	0	0	0	0.471	1.414	0	0	0	1.414	0.015	0	0	0	0.054
IC.10473	0	0	0.017	0.112	0	0	0	0.017	0.122	2E-16	0	0	0.017	0.414	0.866	0	0.013	0.003	0.01	0.066
IC.11100	0	0	0	0.033	0	0	0	0	0.177	0.286	0	0	0.153	0.176	1.155	0	0	0.006	0.039	0.094
IC.1125	0	0.013	0	0.072	0	0	0.014	0	0.113	0	1.491	101.3	0.077	0.232	0	0.03	0.014	0.012	0.019	0
IC.1211	0.235	0.045	0	0	0.065	0.065	0.205	1E-16	0.073	0.291	0.218	0	0.06	0.314	2.828	0.038	0.008	0.004	0.007	0.036
IC.1240	0.06	0.08	0	0.017	0	0.217	0.4	0	0.017	2E-16	0.501	0.346	0	0.353	1.732	0.029	0.035	0.016	0.032	0.099
IC.1260	0.025	0	0	0.109	0	0.025	0	0	0.177	0	0.259	0	0	0.109	1.414	0.031	0.007	0	0.021	0.033
IC.1329	0.182	0.042	0	0	0	0.1	0.489	0	0.133	2E-16	1.732	1	0.108	1	0.346	0.039	0.009	0.023	0.018	0.004
IC.1331	0.031	0	0	0.032	0.049	0.156	0	0	0.031	0.047	2	0	0	0.845	0	0.057	0.008	0.021	0.008	0.181
IC.1378	0	0.011	0	0	0	0	0.011	0	0	0.866	0.866	0.994	0.217	0.917	0.866	0.031	0.005	0.02	0.021	0.136
IC.1386	0.027	0.045	0	0	0.109	0.027	0.24	0	0	0.109	74.95	0.431	0.157	0	1.414	0.012	0.029	0.023	0.008	0.024
IC.193	0	0	0	0	0	0	0	0	0	0	0.346	1.732	2E-16	0	0	4E-04	0.007	0	0	2E-16
IC.2	0	0.108	0	0.074	0	0.247	0.062	1E-16	0.057	0.346	0	0.46	0	0.071	0.866	0.021	0.023	0.005	0.057	0.131
IC.20476	0.141	0.018	0	0	0	0.141	0.018	0	0	0	0.595	0.018	0.202	0.849	0	0.029	0.016	0.018	0.04	0.205
IC.206	0	0.031	0.06	0	0	0	0.171	1.414	0	0	0	1.414	0.225	0.471	0.471	0.026	0.015	0.016	0.031	0.015
IC.239	0.051	0	0	0.038	0	0.053	0.217	0	0.117	2E-16	0.308	2E-16	2E-16	0.867	2E-16	0.017	0.003	0.022	0.032	0
IC.241	0	0.035	0	0	0	0	1.414	0	0	0	0.471	0.035	0	0	0	0.055	0.042	0	0	0
IC.246	0.069	0.013	0	0.098	0	0.069	0.013	0	0.182	0	0.628	0.337	0.133	0.866	0	0.073	0.022	0.002	0.052	0.002
IC.253	0	0.035	0	0	0.074	0	0.035	0	0	0.404	0	0.035	0	0	26.87	0	0.008	0.029	0.049	0.045
IC.277	0.095	0	0	0	0.043	0.12	0	0	0	0.044	0.913	0	0.106	1.491	0.743	0.008	0	0.014	0.024	0.126
IC.295	0	0.019	0	0.109	0	0	0.019	0	0.094	0	0	1.414	0.129	0.372	0	0	0.01	0.01	0.072	0
IC.30048	0	0	0	0.019	0	0.154	0.204	0	0.13	0	0	0	0.118	0.77	0	0.045	0.008	0.015	0.033	0
IC.30281	0	0	0	0	0.06	1E-16	0.157	0	0	0.062	0	0.247	0	0.217	0	0	0.007	0	0.002	0.023
IC.35	0	0.012	0	0.103	0.182	0.124	0.351	0	0.1	0.899	1.732	0.917	0	15.02	1.732	0.032	0.032	0.01	0.059	0.118
IC.387	0	0	0	0	0	2E-16	0	0	0	0	0	0	0	2E-16	0	2E-16	0.003	0	1E-16	1E-16
IC.389	0	0	0	0.037	0.102	0	2E-16	0	0.038	0.108	0	0	2E-16	0.474	0.551	0	0.007	0	0.049	0.06
IC.404	0.028	0.043	0	0	0	0.028	0.201	0	0	2E-16	0.401	0.127	0	0	2E-16	0.009	0.013	0	1E-16	0.015
IC.450	0.022	0	0	0.019	0	0.023	0.346	0	0.15	0	2.371	0.866	0.247	0.582	1.732	0.024	0.015	0.011	0.039	0.016
IC.451	0.092	0	0	0	0.127	0.275	0	0	0.25	0.25	1.732	0	0	0.866	0.886	0.074	0.003	0.009	0.002	0.12
IC.454	0.125	0.017	0	0.02	0	0.033	0.017	0	0.109	0	0.357	0	0	0.722	0	0.063	0.012	0.038	0.047	0
IC.461	0.104	0.052	0	0.18	0.091	0.066	0.201	0	0.192	0.347	5.991	0.529	0.266	0.936	3.432	0.099	0.06	0.027	0.07	0.188
IC.465	0.031	0	0	0	0	0.031	0	0	0	0	0	0	0.109	0.283	0	0.015	0.01	0.038	0.061	0
IC.472	0	0	0	0	0	0	0	0	0	0	0	0	0	0	0	0.02	0	0	0	0
IC.479	0	0	0	0	0.133	0	2E-16	0	0.124	0.411	0	0.433	0.217	0.217	0.866	0.024	0.027	0.014	0.011	0.093
IC.525	0.021	0.055	0	0	0.061	0.181	0.665	0	0.283	0.061	0.453	1.414	0	0.471	1.414	0.036	0.061	0	0.012	0.056
IC.534	0	0	0	0.016	0.049	0.222	0.559	0	0.017	0.047	0.286	0.4	0	0.275	1.826	0.011	0.032	0	0.034	0.049
IC.606	0.133	0	0	0	0	0.226	0	0	0	0	0.133	0	0	0	1.414	0.049	0	0.005	0	0.024
IC.616	0.063	0	0	0.125	0.056	0.191	2E-16	0	0.116	0.371	0.063	3.464	0.25	1.952	4.093	0.079	0.015	0.022	0.021	0.098
IC.629	0.059	0.022	0	0.029	0	0.244	0.178	0	0.128	0	0.936	0.851	0.222	77.38	0	0.058	0.041	0.02	0.02	0
IC.660	0	0	0	0	0	0	0	1E-16	0.108	2E-16	0	0	0	1	2E-16	0	0	0	0.013	0
IC.665	0.072	0.106	0	0	0.048	0.292	0.449	0.248	0.106	0.513	0.228	1.702	0.16	0.373	0.561	0.06	0.031	0.032	0.029	0.054
IC.684	0.213	0	0	0	0	0.41	0	0	0	0	0	0	0	0.286	0.286	0.035	0	0	0.027	0.026
IC.689	0.027	0.026	0	0	0.253	0.149	0.027	0	0	0.141	2	0.667	0	0	0.672	0.036	0.049	0.013	0	0.06
IC.711	0	0	0	0	0	0	0	0	0.283	0	0	0	0	0	0	0	0	0.006	0.025	0
IC.736	0.127	0	0.015	0.086	0.037	0.037	0	0.015	0.035	0.359	1.369	0.913	0.109	1.225	0.913	0.028	0.015	0.03	0.037	0.081
IC.797	0	0	0	0	0.105	0.128	0	0	0.128	0.182	0.182	2	0.061	0.385	2	0.032	0.009	0.018	0.017	0.037
IC.818	0	0	0	0	0.051	0	2E-16	0	0	0.377	0	0	0	0	0.049	0	0.004	0	0.004	0.112
IC.857	0.094	0.093	0	0.233	0.056	0.089	0.331	0.346	0.09	0.465	0.33	0.088	0.346	0.911	1.732	0.114	0.044	4E-04	0.046	0.039
IC.912	0	0	0	0	0	0	0	0	0	0.385	0	0	0	0	1.155	0	0.003	0.015	0.003	0.095
IC.940	0	0	0	0	0	0	0	0	0	0	0	0	0	0	0	0	0.004	0.006	0	0
IC.999	0.02	0	0	0	0	0.112	0	0	0	0	1.155	0	0	2	0	0.056	0	0.016	0.011	0.014
IC.JCP	0	0.056	0.017	0	0.047	0.217	0.057	0.017	0.108	0.379	1.732	0.61	0.108	0.247	0.046	0.053	0.008	0.005	0.04	0.053
IC.JJM	0	0	0	0	0	0	0	0	0.203	0	0.248	0.391	0	0.228	0	0.018	0.006	0.007	0.011	0
IC.REH	0.081	0	0	0	0	0.13	0	0	0	0	0.088	0.667	0	0	0	0.086	0.006	0	0.004	0
