# Supplementary material for: Accurate predictions of population-level changes in sequence and structural properties of HIV-1 Env using a volatility-controlled diffusion model
Source: PLoS Biol. 2017 Apr 6;15(4):e2001549. doi: 10.1371/journal.pbio.2001549 (PMC5383018; doi:10.1371/journal.pbio.2001549)
Supplement: S13 Fig — An example is given of calculating the likelihood of Alanine appearing at a given position when the reference sequence contained Proline. Volatility of charge, molecular weight and hydropathy was calculated for the position. For convenience, molecular weight was converted to a scale of 0 to 1. For each feature type, a probability density function was generated that describes the likelihood of change to each amino acid based on the measured Volatility of the feature at this position, the ancestral state (i.e., Proline) and query (i.e., Alanine in this example). The combined likelihood based on Volatilities of the three feature types was calculated as the product of the three functions. (PDF) [file pbio.2001549.s013.pdf]

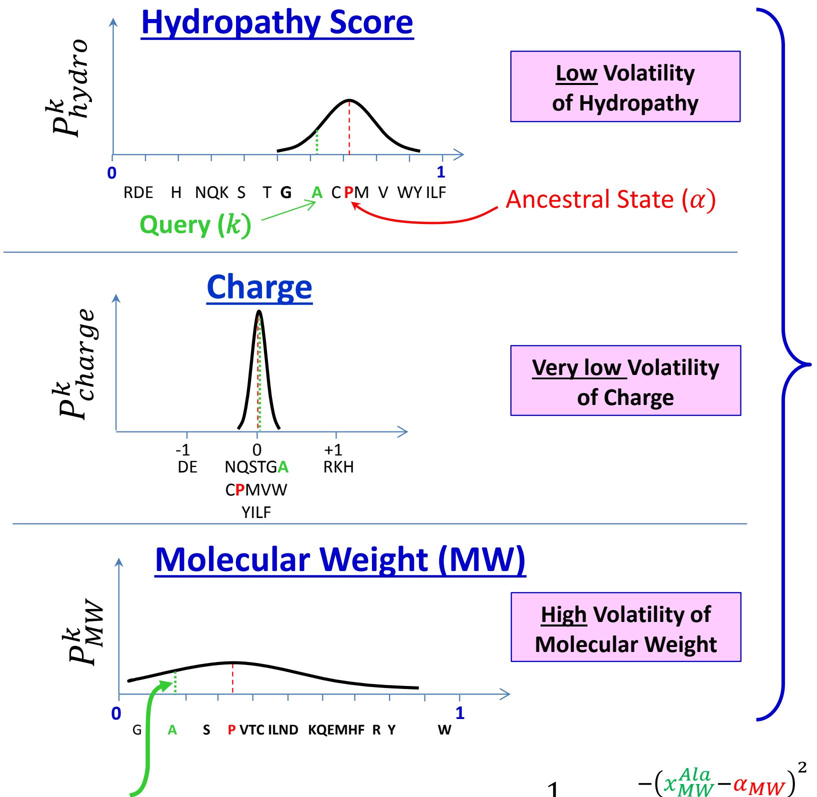

$$P_{Total}^{Ala} = P_{hydro}^{Ala} \cdot P_{charge}^{Ala} \cdot P_{MW}^{Ala}$$

$$P_{MW}^{Ala} = f(x_{MW} \mid \alpha_{MW}, V_{MW}) = \frac{1}{\sqrt{2\pi V_{MW}}} e^{\frac{-\left(x_{MW}^{Ala} - \alpha_{MW}\right)^2}{2V_{MW}}}$$
