# Supplementary material for: Accurate predictions of population-level changes in sequence and structural properties of HIV-1 Env using a volatility-controlled diffusion model
Source: PLoS Biol. 2017 Apr 6;15(4):e2001549. doi: 10.1371/journal.pbio.2001549 (PMC5383018; doi:10.1371/journal.pbio.2001549)
Supplement: S14 Fig — Data describe Envs isolated from all longitudinal and cross-sectional plasma samples collected in Iowa City and Seattle. Data underlying this figure can be found in S6 Data. (PDF) [file pbio.2001549.s014.pdf]

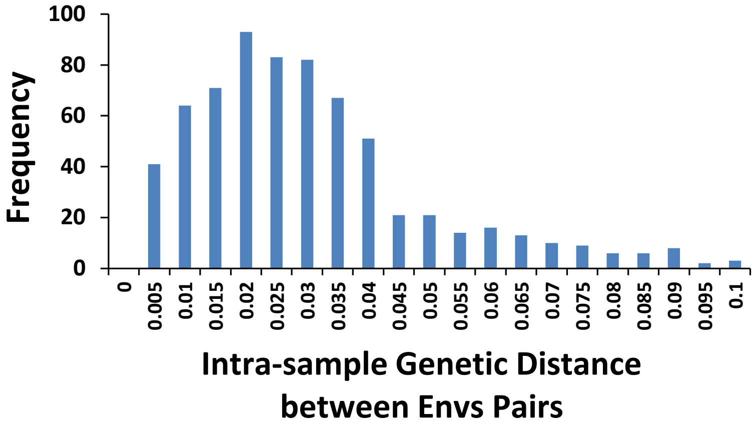

Number of values	682
Minimum	0.00115
Median	0.02423
Maximum	0.1103
Mean	0.02826
Std. Deviation	0.01951
Lower 95% CI of mean	0.02679
Upper 95% CI of mean	0.02972

D'Agostino & Pearson omnibus normality test

K^2	142
P value	< 0.0001
Passed normality test (alpha=0.05)?	No
